# The Effects of mHealth Interventions on Quality of Life, Anxiety, and Depression in Patients With Coronary Heart Disease: Meta-Analysis of Randomized Controlled Trials

**DOI:** 10.2196/52341

**Published:** 2024-06-11

**Authors:** Qiao Ling Hou, Le Yang Liu, Ying Wu

**Affiliations:** 1 School of Nursing Capital Medical University Beijing China

**Keywords:** mobile health, coronary heart disease, quality of life, anxiety, depression, meta-analysis, mobile phone

## Abstract

**Background:**

Coronary heart disease (CHD) is the leading cause of death globally. In addition, 20% to 40% of the patients with CHD have comorbid mental health issues such as anxiety or depression, affecting the prognosis and quality of life (QoL). Mobile health (mHealth) interventions have been developed and are widely used; however, the evidence for the effects of mHealth interventions on QoL, anxiety, and depression in patients with CHD is currently ambiguous.

**Objective:**

In this study, we aimed to assess the effects of mHealth interventions on QoL, anxiety, and depression in patients with CHD.

**Methods:**

We searched the Cochrane Library, PubMed, Embase, CINAHL, Web of Science, China National Knowledge Infrastructure, and Wanfang databases from inception to August 12, 2023. Eligible studies were randomized controlled trials that involved patients with CHD who received mHealth interventions and that reported on QoL, anxiety, or depression outcomes. We used the Cochrane risk-of-bias tool for randomized trials to evaluate the risk of bias in the studies, ensuring a rigorous and methodologically sound analysis. Review Manager (desktop version 5.4; The Cochrane Collaboration) and Stata MP (version 17.0; StataCorp LLC) were used to conduct the meta-analysis. The effect size was calculated using the standardized mean difference (SMD) and its 95% CI.

**Results:**

The meta-analysis included 23 studies (5406 participants in total) and showed that mHealth interventions significantly improved QoL in patients with CHD (SMD 0.49, 95% CI 0.25-0.72; Z=4.07; *P*<.001) as well as relieved their anxiety (SMD −0.46, 95% CI −0.83 to −0.08; Z=2.38; *P*=.02) and depression (SMD −0.34, 95% CI −0.56 to −0.12; Z=3.00; *P*=.003) compared to usual care. The subgroup analyses indicated a significant effect favoring the mHealth intervention on reducing anxiety and depressive symptoms compared to usual care, especially when (1) the intervention duration was ≥6 months (*P*=.04 and *P*=.001), (2) the mHealth intervention was a simple one (only 1 mHealth intervention was used) (*P*=.01 and *P*<.001), (3) it was implemented during the COVID-19 pandemic (*P*=.04 and *P*=.01), (4) it was implemented in low- or middle-income countries (*P*=.01 and *P*=.02), (5) the intervention focused on mental health (*P*=.01 and *P*=.007), and (6) adherence rates were high (≥90%; *P*=.03 and *P*=.002). In addition, comparing mHealth interventions to usual care, there was an improvement in QoL when (1) the mHealth intervention was a simple one (*P*<.001), (2) it was implemented in low- or middle-income countries (*P*<.001), and (3) the intervention focused on mental health (*P*<.001).

**Conclusions:**

On the basis of the existing evidence, mHealth interventions might be effective in improving QoL and reducing anxiety and depression in patients with CHD. However, large sample, high-quality, and rigorously designed randomized controlled trials are needed to provide further evidence.

**Trial Registration:**

PROSPERO CRD42022383858; https://tinyurl.com/3ea2npxf

## Introduction

### Background

Coronary heart disease (CHD) is the leading cause of death globally, taking an estimated 8.9 million lives each year, representing 16% of all deaths [1-4]. The prevalence of psychological disorders in patients with CHD is higher than that in the general population according to mounting evidence, and CHD morbidity and mortality are correlated with mental health status [5,6]. According to earlier research, 20% to 40% of the patients with CHD have concurrent mental health issues, such as anxiety and depression [7,8], which are not only risk factors for inducing or exacerbating CHD but also directly affect the prognosis, increase the risk of recurring major adverse cardiac events and death by reducing patients’ compliance with treatment, and greatly reduce the clinical benefits of treatment [9]. Meanwhile, comorbid anxiety and depression are associated with diminished quality of life (QoL) [10,11].

A study involving 190 patients with CHD who were followed for 36 months found that the incidence of anxiety and depression increased over time, with anxiety increasing persistently from 42.6% to 51.1% and depression increasing from 33.3% to 43.7% [5]. Therefore, the European Society of Cardiology and the American Heart Association guidelines on the secondary prevention of CHD make explicit recommendations for alleviating psychological problems such as anxiety or depression in patients with CHD [3,12]. Furthermore, to improve well-being, supportive interventions are required to assist patients in dealing with challenges related to their QoL, anxiety, and depression.

Currently, guidelines state that patients with CHD with anxiety and depression should receive increased attention and assistance to enhance adherence to lifestyle changes, engage in psychotherapy, and receive pharmacological therapies [3]. Evidence suggests that healthy behaviors such as quitting smoking, increasing physical activity, and improving diet are beneficial and applicable to patients with CHD with mental issues [13-15]. Psychotherapy, such as psychological counseling and cognitive behavioral therapy, can improve patients’ self-efficacy and lead to a healthy lifestyle, reducing the risks of CHD recurrence and mortality [16,17]. However, these methods are time consuming and labor intensive, and they lack sustainability, which makes it difficult to meet the needs of patients and promote well-being [18]. In addition, evidence indicates that patients with CHD with moderate to severe major depression should be considered for antidepressive treatment with a selective serotonin reuptake inhibitor, which can lower the rates of CHD readmission and all-cause mortality [19], but it can have some side effects [20].

Hence, there is a need for sustainable, time-saving, and effective ways to improve patients’ QoL as well as reduce their anxiety and depression. The exponential growth and availability of mobile health (mHealth) technologies may offer a fresh stress management strategy [21]. The World Health Organization describes mHealth as a subfield of eHealth that involves the delivery of health services and information via mobile technology such as mobile phones and PDAs [22,23].

### The Effectiveness of mHealth Interventions

There is rising evidence of the effectiveness of mHealth interventions on QoL, anxiety, and depression in patients with CHD. However, the evidence is often inconsistent or even conflicting. The studies by Yudi et al [24] and Houchen-Wolloff et al [25] found that, compared to usual care, mHealth interventions did not effectively relieve anxiety and depressive symptoms in patients with CHD. By contrast, the results of other studies showed that the mHealth group significantly outperformed the control group in terms of QoL and psychological distress [26,27]. The disparate conclusions may be due to the fact that there are still questions and doubts about the heterogeneity of the effects of mHealth interventions across various intervention contents, such as different intervention methods, durations, and adherence rates. This lack of clear understanding about how these factors influence outcomes limits the availability of definitive evidence regarding the psychological care of patients with CHD.

Hence, our meta-analysis aims to investigate the effectiveness of mHealth interventions on QoL, anxiety, and depression outcomes in patients with CHD.

## Methods

### Study Design

The study protocol was registered on PROSPERO (CRD42022383858), and we followed the PRISMA (Preferred Reporting Items for Systematic Reviews and Meta-Analyses) guidelines [28,29] (Multimedia Appendix 1).

### Search Strategy

We conducted a thorough literature search to identify randomized controlled trials (RCTs) related to mHealth interventions for patients with CHD. The databases searched were Cochrane Library, PubMed, Embase, CINAHL, Web of Science, China National Knowledge Infrastructure, and Wanfang (Multimedia Appendix 2). In addition, we manually reviewed the reference lists of the included full texts, performed a supplementary search on the ClinicalTrials.gov trial registration platform, and contacted the authors for further information if needed.

The search terms were constructed by combining subject words with free words. The key terms included *mobile health*, *mHealth*, *eHealth*, *telehealth*, *telemedicine,* *digital health*, *mobile phone*, *cellphone*, *smartphone*, smartphone app*, cellphone app*, mobile app*, *smartphone-based*, *cellphone-based*, *mobile phone-based*, portable electronic app*, portable software app* and *coronary disease*, *coronary heart disease*, *coronary artery disease*, *myocardial ischemia*, *myocardial revascularization*, *acute coronary syndrome*, *coronary artery bypass*, *percutaneous coronary intervention*.

### Eligibility Criteria

The eligibility criteria were structured according to population, intervention, comparison, outcomes, and study design (PICOS).

- Population: adults (aged ≥18 years) with CHD, including patients with myocardial infarction, revascularization (stent, coronary artery bypass grafting, or percutaneous transluminal coronary angioplasty), or CHD confirmed by angiography
- Intervention: mHealth interventions (eg, mobile based or web based monitoring devices, PDAs, or wireless devices; no restriction on duration, frequency, or type of intervention)
- Comparison: comparison group did not receive mHealth intervention; instead, it received a different intervention or placebo
- Outcomes: studies that reported QoL, anxiety, or depression as outcomes
- Study design: only RCTs (no restrictions on language; studies in languages other than English and Chinese would be translated using translation software)

### Exclusion Criteria

Studies were excluded if (1) after contacting the authors, the complete text was unavailable, and important data could not be extracted; (2) the outcomes were unclear, and the data could not be combined, transformed, and used for analysis; (3) they were duplicate publications; or (4) they were pilot studies.

### Study Selection

All studies retrieved from the database search were managed and organized by exporting the citations to EndNote X9 (Clarivate) and Rayyan (a web application used to screen a large number of records for rapid selection; Rayyan Systems Inc) [30]. Microsoft Excel was used for data extraction. Two reviewers (QLH and LYL) screened the titles, abstracts, and full texts of studies independently. Disagreements between the reviewers regarding the title and abstract screening, full-text review, and reasons for exclusion were resolved by discussion with the third reviewer (YW).

### Data Extraction

A prespecified electronic data extraction table following the PRISMA guidelines [28,29] was designed to extract (1) study information (author, year, country of origin, sample size, intervention methods, frequency, duration, and adherence rates), (2) primary and secondary outcomes as well as measurement scales, and (3) key information for the risk-of-bias assessment. The data extraction table was pilot-tested by QLH with 3 studies (reviewed by LYL). Next, authors QLH and LYL retrieved data from the full-text papers, and conflicts were resolved by discussion and consultation with the third author (YW).

### Risk of Bias Assessment

Two reviewers (QLH and LYL) independently assessed the quality of studies according to the Cochrane Handbook for Systematic Reviews of Interventions (version 6.3) [31], which includes 7 domains: (1) random sequence generation (selection bias), (2) allocation sequence concealment (selection bias), (3) blinding of participants and personnel (performance bias), (4) blinding of outcome assessment (detection bias), (5) incomplete outcome data (attrition bias), (6) selective outcome reporting (reporting bias), and (7) other sources of bias. The criteria for low, unclear, and high risk of bias within and across studies followed the Cochrane Handbook for Systematic Reviews of Interventions (version 6.3) [31]. A quality grade of *A* (low bias) was assigned if 7 domains were all low risk of bias, *B* (moderate bias) if ≥1 domains were unclear or high risk of bias, and *C* (high bias) if none of the 7 domains were satisfied. Disagreements between the 2 reviewers were settled by discussion with the third reviewer (YW). The ultimate decision was taken by consensus of the 3 reviewers.

### Data Synthesis and Statistical Analysis

The meta-analysis was conducted using Review Manager (desktop version 5.4; The Cochrane Collaboration) and Stata MP (version 17.0; StataCorp LLC). We selected standardized mean difference (SMD) and 95% CI as the effect indicators because the QoL, anxiety, and depression were measured using different scales in different studies. Heterogeneity was evaluated using the *I*^2^ statistic and defined as *not important* (*I*²≤25%), *moderate* (*I*²=26%-50%), *substantial* (*I*²=51%-75%), and *considerable* (*I*²>75%) [32]. Depending on the *I*^2^ values, a random effects model (*I*^2^≥50%) or a fixed effects model (*I*^2^<50%) was selected.

To investigate the heterogeneity across several subgroups, subgroup analyses were performed for the different intervention durations (<6 months or ≥6 months), different intervention methods (simple intervention or complex intervention), the influence of the COVID-19 pandemic (before or during the pandemic), different country types (high-income country [HIC] or low- or middle-income country [LMIC]), different intervention contents (whether the mHealth intervention focused on mental health), and different levels of adherence (≥90% or <90%) to explore the heterogeneity among several subgroups. A simple mHealth group was defined as one that used only 1 mHealth intervention, such as a WeChat message or an SMS text message, while a complex mHealth group was defined as one that used multiple mHealth interventions, such as simultaneous use of smartphone apps and wearable devices. Different levels of adherence were defined as *higher* (intervention group adherence rate was ≥90%) and *lower* (intervention group adherence rate was <90%).

All statistical tests were 2-tailed, and *P*<.05 was considered statistically significant. We used funnel plots to evaluate publication bias, and plot asymmetry was tested using the Begg and Egger tests in Stata MP (version 17.0). The leave-one-out strategy was used to find outliers and analyze their influences. Moreover, the trim-and-fill method developed by Duval and Tweedie [33] was used to eliminate any tiny studies with substantial effect sizes from the positive side of the funnel plot to further clarify the potential impact of publication bias. In addition, a sensitivity analysis was carried out by redoing the meta-analysis after eliminating papers that had a high probability of bias [34]. The data used in the meta-analysis are all postintervention data.

## Results

### Overview

**Figure 1:**
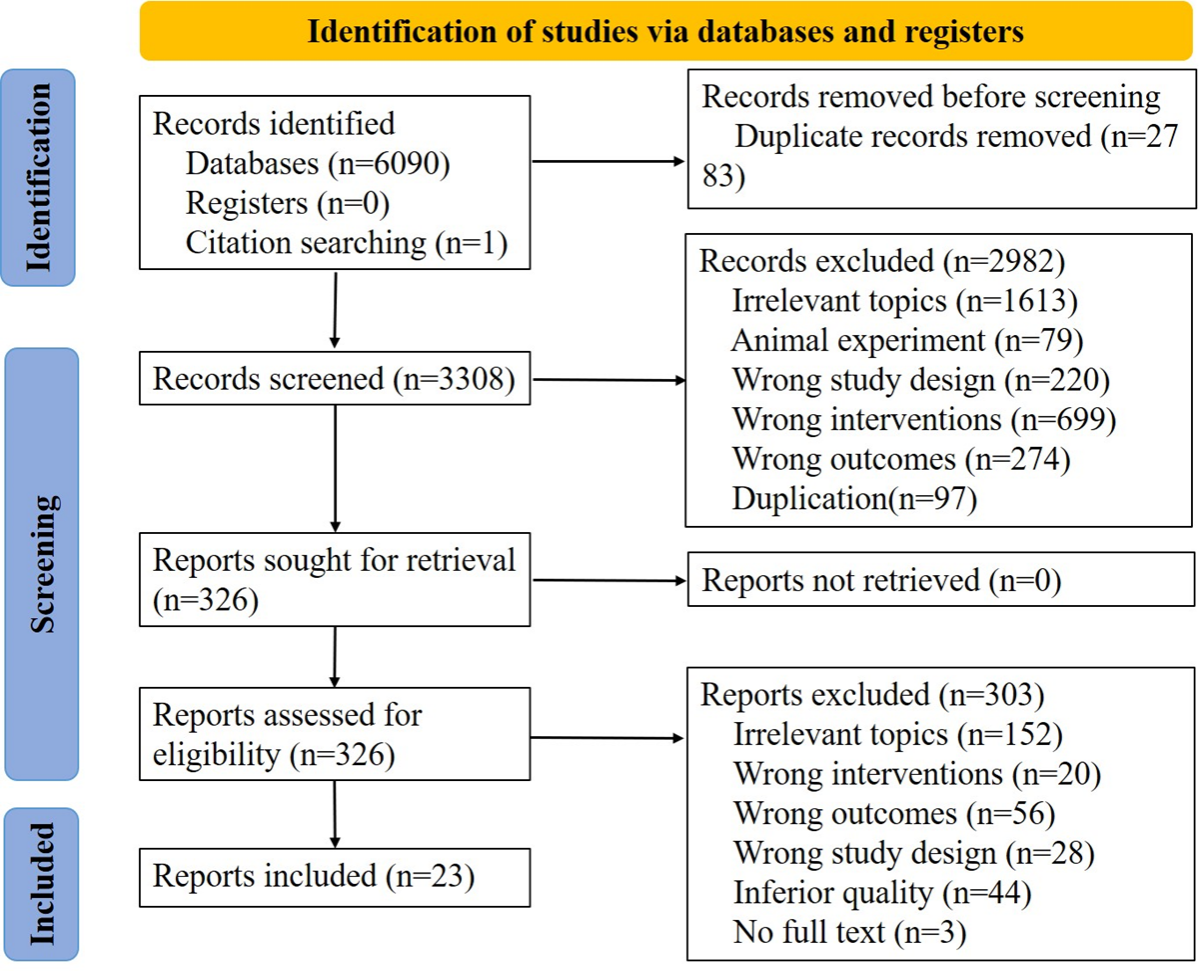
Flowchart of the literature search. The databases searched were the Cochrane Library (n=1110), Web of Science (n=1484), PubMed (n=448), Embase (n=555), CINAHL (n=1608), China National Knowledge Infrastructure (n=453), and Wanfang (n=432).

Figure 1 depicts the meta-analysis study selection process. On the basis of the search terms we defined, our initial search generated 6091 publications. Of these 6091 publications, we excluded 2783 (45.69%) duplicates and irrelevant publications. Of the remaining 3308 publications, we excluded 2982 (90.15%) after abstract screening. Of the 326 full-text reports obtained, we excluded 303 (92.9%) on the basis of the selection criteria and included 23 (7.1%) in the analysis. Of these 23 studies, 19 (83%) were in English and 4 (17%) in Chinese; they were all published between 2015 and 2023.

### Characteristics of the Included Studies

#### Participants

The 23 RCTs selected [24-27,35-53] included 5406 participants (n=2708, 50.09% participants in the intervention groups and n=2698, 49.91% in the control groups), with sample sizes per study varying from 51 to 1424. All patients with CHD in the 23 RCTs were in the cardiac rehabilitation (CR) stage, including those who had undergone revascularization procedures (such as coronary artery bypass grafting or percutaneous transluminal coronary angioplasty) as well as patients with CHD confirmed through angiography. The RCTs were conducted in Asia (12/23, 52%) [27,37-41,43,45,48-51], Europe (6/23, 26%) [25,26,35,42,44,47], and Australia and New Zealand (5/23, 22%) [24,36,46,52,53]. Of the 23 studies, 19 (83%) reported QoL, 13 (57%) reported anxiety, and 17 (74%) reported depression (Tables 1 and 2).

**Table 1:** Characteristics of included studies (n=23).

Study and year	Country	Participants	Stage of disease	Sample	IG^a^	CG^b^	Retention rate, n/N (%)
Batalik et al [35], 2020	Czech Republic	Aged >18 years, diagnosed with CVD^c^ (angina pectoris, myocardial infarction in the last 6 months, with left ventricular ejection fraction >45%)	Telerehabilitation	51 (IG: 25; CG: 26)	Wrist heart rate monitor	Usual care	IG: 25/28 (89.3); CG: 26/28 (92.9)
Cheung et al [53], 2023	Australia	CHD^d^ was defined as previous myocardial infarction or documented >50% occlusion of a major coronary artery on coronary angiography	Previous myocardial infarction	453 (IG: 228; CG: 225)	Semipersonalized SMS text messaging service	Usual care	IG: 228/256 (89.1); CG: 225/250 (90)
Chow et al [36], 2022	Australia	Diagnosis of ACS^e^	Secondary prevention	1424 (IG: 716; CG: 708)	SMS text messaging (customized and personalized)	Usual care	IG: 697/716 (97.3); CG: 682/708 (96.3)
Dalli Peydró et al [26], 2022	Spain	Low-risk ACS, left ventricular ejection fraction ≥50%	Cardiac telerehabilitation	59 (IG: 31; CG: 28)	Monitoring of heart rate via participant smartphone and heart rate monitor	Usual care	IG: 31/33 (93.9); CG: 28/34 (82.4)
Dorje et al [37], 2019	China	Aged ≥18 years with documented CHD (including myocardial infarction and unstable or stable angina)	CR^f^ and secondary prevention	312 (IG: 156; CG: 156)	Smartphone-based home-based CR (individualized exercise prescription and remote supervision)	Usual care	IG: 134/156 (85.9); CG: 131/156 (84)
Duan et al [38], 2018	China	Aged between 18 and 75 years, CHD	Home-based CR	114 (IG: 60; CG: 54)	8-week web-based intervention	Usual care	IG: 44/60 (73.3); CG: 39/54 (72.2)
Fang et al [39], 2019	China	Patients with low risk after PCI^g^	CR	67 (IG: 33; CG: 34)	Real-time physiological monitoring; home visits; telephone call	Usual care	IG: 33/40 (82.5); CG: 34/40 (85)
Hisam et al [40], 2022	Pakistan	Patients after ACS (ST-elevation myocardial infarction, non–ST-elevation myocardial infarction, and unstable angina)	CR	160 (IG: 80; CG: 80)	Diurnal mobile texting (standardized messages about healthy lifestyle changes through app)	Standard care	IG: 70/80 (87.5); CG: 49/80 (61.3)
Houchen-Wolloff et al [25], 2018	United Kingdom	Confirmed primary diagnosis of CHD (including angina, after myocardial infarction and after PCI	CR	60 (IG: 37; CG: 23)	Web-based CR program	Usual care	IG: 33/37 (89.2); CG: 21/23 (91.3)
Huang et al [41], 2017	China	Patients with unstable angina pectoris and undergoing their first elective PCI	CR	64 (IG: 31; CG: 33)	WeChat	Usual care	IG: 31/31 (100); CG: 33/33 (100)
Johnston et al [42], 2016	Sweden	Women or men aged >18 years, diagnosed as having an ST-elevation myocardial infarction or a non–ST-elevation myocardial infarction	Secondary prevention stage	151 (IG: 85; CG: 77)	Web-based application (interactive and personalized)	Simplified drug adherence diary	IG: 85/91 (93.4); CG: 77/83 (92.8)
Kang et al [43], 2021	Korea	Clinical ASCVD^h^	Secondary prevention stage	643 (IG: 322; CG: 321)	A smartphone app (personalized)	Usual care	IG: 322/333 (96.7); CG: 321/333 (96.4)
Kang G et al [27], 2023	China	Participants were required to be aged ≥18 years and to have a diagnosis of stable CAD^i^	Secondary prevention stage	200 (IG: 100; CG: 100)	WeChat	Usual care	IG: 98/100 (98); CG: 98/100 (98)
Kraal et al [44], 2017	Netherlands	Patients who entered CR after ACS (myocardial infarction or unstable angina) or a revascularization procedure (PCI or coronary artery bypass grafting)	Home-based CR	78 (IG: 37; CG: 41)	Heart rate monitor and web application	Usual care	IG: 37/45 (82.2); CG: 41/45 (91.1)
Pakrad et al [45], 2021	Iran	Patients who have undergone coronary artery bypass grafting	CR	88 (IG: 44; CG: 44)	CR was delivered in person in the first month; over the following 3 months, it was delivered via smartphone	Usual care	IG: 42/44 (95.5); CG: 39/44 (88.6)
Pfaeffli Dale et al [52], 2015	New Zealand	CHD (myocardial infarction, angina, or revascularization)	CR	123 (IG: 61; CG: 62)	Personalized SMS text messaging service, supporting website, and pedometer	Usual care	IG: 57/61 (93.4); CG: 59/62 (95.2)
Shariful Islam et al [46], 2019	Australia	CHD was defined as myocardial infarction, coronary artery bypass grafting, or PCI or proven angiographically	Secondary prevention	883 (IG: 333; CG: 350)	SMS text messaging	Usual care	IG: 333/352 (94.6); CG: 350/358 (97.8)
Snoek et al [47], 2021	Netherlands	ACS, PCI, or coronary artery bypass grafting within 3 months before the start of the CR program	CR	122 (IG: 61; CG: 61)	Smartphone and Bluetooth-connected heart rate belt	Usual care	IG: 59/61 (96.7); CG: 59/61 (96.7)
Su and Yu [48], 2021	China	Initial diagnosis of CHD based on angiography or the exacerbation of CHD in previously diagnosed cases	Nurse-led eHealth CR	146 (IG: 73; CG: 73)	Nurse-led eHealth CR platform and WeChat platform	Usual care	IG: 66/73 (90.4); CG: 58/73 (79.5)
Wang [49], 2018	China	Patients aged ≥18 years, and coronary artery stenosis indicated by first coronary angiography and successfully implanted one or more stents	Postdischarge stage	51 (IG: 23; CG: 28)	Mobile app	Usual care	IG: 23/32 (71.9); CG: 28/32 (87.5)
Yudi et al [24], 2021	Australia	Patient over 18 years old with a diagnosis of an ACS and documented coronary artery disease on angiography (coronary artery stenosis >50%)	Cardiac rehabilitation	168 (IG: 83; CG: 85)	Adjunctive smartphone-based CR program (SCRP)	Usual care	IG: 83/103 (80.6); CG: 85/103 (82.5)
Zhang et al [50], 2020	China	Patients who underwent a first and successful PCI procedure	Continuous care	88 (IG: 44; CG: 44)	Mobile app	Usual care	IG: 44/44 (100); CG: 44/44 (100)
Zheng [51], 2021	China	Patients undergoing initial PCI for CAD	Continuous care	90 (IG: 45; CG: 45)	Follow-up continuous nursing intervention	Usual care	IG: 45/45 (100); CG: 45/45 (100)

^a^IG: intervention group.

^b^CG: control group.

^c^CVD: cardiovascular disease.

^d^CHD: coronary heart disease.

^e^ACS: acute coronary syndrome.

^f^CR: cardiac rehabilitation.

^g^PCI: percutaneous coronary intervention.

^h^ASCVD: atherosclerotic cardiovascular disease.

^i^CAD: coronary artery disease.

**Table 2:** Characteristics of included studies (n=23).

Study and year	Intervention frequency	Intervention duration (months)	Adverse events	Outcomes	Scales
Batalik et al [35], 2020	3 times a week	3	Remotely monitored telerehabilitation seems to be safe, and no adverse cardiac events occurred during the intervention	A^a^	SF-36^b^
Cheung et al [53], 2023	4 messages a week	6	No adverse events or deaths were deemed related to the intervention	A and B^c^	SF-12^d^ and PHQ-9^e^
Chow et al [36], 2022	4 messages a week in the first 6 months and 3 messages a week over the subsequent 6 months	12	The intervention was safe	A, B, and C^f^	SF-12, PHQ-9, and GAD-7^g^
Dalli Peydró et al [26] 2022	Daily	10	During the study period, 3 patients were readmitted, but the readmissions were not related to the intervention	A, B, and C	EQ-5D-5L and HADS^h^
Dorje et al [37], 2019	First and second month: 4 times per week; from third to sixth month: twice a week	6	No adverse events or SMART-CR/SP^i^ program-related safety issues were recorded during the study	A, B, and C	SF-12 and PHQ-9
Duan et al [38], 2018	Once a week	2	Not mentioned	A and B	WHOQOL^j^ and CES-D^k^
Fang et al [39], 2019	3 times a week	1.5	Not mentioned	A and B	SF-36 and CDS^l^
Hisam et al [40], 2022	Daily	6	Not mentioned, but the intervention in this study was a medically supervised cardiac rehabilitation program	A	SF-12 and MacNew^m^
Houchen-Wolloff et al [25], 2018	Daily	6	There were 2 adverse events in the web group and 4 in the control group, but all were unrelated to the study	A, B, and C	MacNew and HADS
Huang et al [41], 2017	Every Monday and Thursday	3	Not mentioned	A	SAQ^n^
Johnston et al [42] 2016	Daily	6	No adverse device effects were reported during the study	A	EQ-5D VAS
Kang et al [43], 2021	Daily	6	Not mentioned	B	BDI^o^
Kang G et al [27], 2023	3 times a week	12	There were no obvious differences between the 2 groups in terms of adverse events	A, B, and C	SAQ, HAMA^p^, and HAMD^q^
Kraal et al [44], 2017	At least 2 training sessions a week, and once a week, the patient received feedback	3	No serious adverse events were recorded during center-based and home-based training	A, B, and C	MacNew and HADS
Pakrad et al [45], 2021	8 in-person sessions and 4 group discussion sessions; in addition, each participant was contacted 24 times over 3 months through the mobile app	4	None of the intervention participants were hospitalized	A, B, and C	SF-36 and DASS-21^r^
Pfaeffli Dale et al [52], 2015	7 messages per week for 12 weeks, followed by 5 messages per week for the next 12 weeks	6	There were 13 serious adverse events reported (intervention: n=8; control: n=5), but none were study related	B and C	HADS
Shariful Islam et al [46], 2019	4 times a week	6	Not mentioned	A and B	SF-12 and PHQ-9
Snoek et al [47], 2021	5 days a week	6	No cardiovascular mortality and near sudden cardiac death was registered	A, B, and C	MacNew and HADS
Su and Yu [48], 2021	Daily	3	Not mentioned	A, B, and C	MacNew and DASS-21
Wang et al [49], 2018	Daily	6	Not mentioned	A	SAQ
Yudi et al [24], 2021	Daily	2	Not mentioned	A, B, and C	SF-36, EQ-5D-5L, HADS, and CDS
Zhang et al [50], 2020	Daily	6	Not mentioned	B and C	SAS^s^ and SDS^t^
Zheng [51], 2021	At 1 week and 1, 3, 6, and 12 months after discharge	12	Not mentioned	A, B, and C	SF-36, SAS, and SDS

^a^A: health-related quality of life.

^b^SF-36: Short Form Health Survey-36.

^c^B: depression.

^d^SF-12: Short Form Health Survey-12.

^e^PHQ-9: Patient Health Questionnaire-9.

^f^C: anxiety.

^g^GAD-7: General Anxiety Disorder-7.

^h^HADS: Hospital Anxiety and Depression Scale.

^i^SMART-CR/SP: smartphone- and social media–based cardiac rehabilitation and secondary prevention.

^j^WHOQOL: World Health Organization Quality of Life.

^k^CES-D: Center for Epidemiologic Studies Depression Scale.

^l^CDS: Cardiac Depression Scale.

^m^MacNew: MacNew Heart Disease Health-Related Quality of Life.

^n^SAQ: Seattle Angina Questionnaire.

^o^BDI: Beck Depression Inventory.

^p^HAMA: Hamilton Anxiety Scale.

^q^HAMD: Hamilton Depression Scale.

^r^DASS-21: Depression, Anxiety, and Stress Scale-21.

^s^SAS: Self-Rating Anxiety Scale.

^t^SDS: Self-Rating Depression Scale.

#### The Intervention Groups

In the 23 studies, the main mHealth intervention methods used were SMS text messaging (4/23, 17%), WeChat (4/23, 17%), mobile apps (11/23, 48%), wearable devices (5/23, 22%), and web-based interventions (5/23, 22%). The mHealth intervention durations ranged from 6 weeks to 12 months; of the 23 studies, 8 (35%) had intervention durations <6 months, and 15 (65%) had intervention durations ≥6 months. Of the 23 studies, 16 (70%) used only 1 mHealth intervention, whereas 7 (30%) used ≥2 mHealth interventions simultaneously. Of the 23 studies, 18 (78%) were conducted and completed before the outbreak of the COVID-19 pandemic, whereas 5 (22%) were conducted during the COVID-19 pandemic; furthermore, 12 (52%) were conducted in HICs and 11 (48%) in LMICs. In 9 (39%) of the 23 studies, the mHealth interventions focused on mental health, whereas in 14 (61%) studies, the mHealth interventions were less relevant to mental health. Of the 23 studies, 10 (43%) have lower (<90%) adherence rates, whereas 13 (57%) have *higher* (≥90%) adherence rates.

#### The Control Groups

The control group received usual care, including routine psychological counseling, health education, paper-based CHD educational booklets, and follow-up after discharge.

#### Outcomes

In sum, of the 23 studies, 19 (83%) reported QoL scores using the Short Form Health Survey-36 (n=5, 26%), the Short Form Health Survey-12 (n=4, 21%), the EQ-5D-5L (n=3, 16%), the MacNew Heart Disease Health-Related Quality of Life scale (n=4, 21%), and the Seattle Angina Questionnaire (n=3, 16%). Anxiety scores were reported by 13 (57%) of the 23 studies. Anxiety was measured with the Hospital Anxiety and Depression Scale (6/13, 46%); the Self-Rating Anxiety Scale (2/13, 15%); the Hamilton Anxiety Scale (1/13, 8%); the Depression, Anxiety, and Stress Scale-21 (2/13, 15%); and the Generalized Anxiety Disorder-7 (2/13, 15%). In addition, 17 (74%) of the 23 studies reported depression scores using the Hospital Anxiety and Depression Scale (n=5, 29%); the Self-Rating Depression Scale (n=2, 12%); the Hamilton Depression Scale (n=1, 6%); the Patient Health Questionnaire-9 (n=4, 24%); the Depression, Anxiety, and Stress Scale-21 (n=1, 6%); the Cardiac Depression Scale (n=2, 12%); the Center for Epidemiologic Studies Depression Scale (n=1, 6%); and the Beck Depression Inventory (n=1, 6%).

#### Risk of Bias in Studies

**Figure 2:**
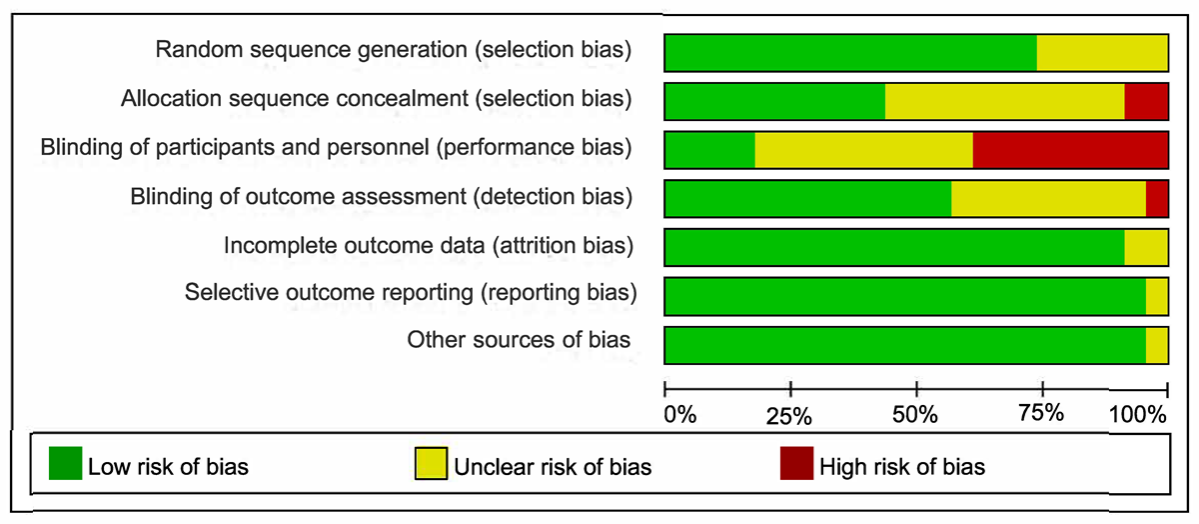
Analysis of the risk of bias.

The Cochrane Handbook for Systematic Reviews of Interventions (version 6.3) was used to evaluate bias [54]. Of the 23 studies, 17 (74%) [25,27,35-37,40,41,43-50,52,53,55] used a computer-generated list of random numbers to allocate individuals to the intervention or control groups, which was deemed minimal risk of bias, and 10 (43%) [26,36,37,40,44-46,48,52,53] concealed the allocation of participants, which was considered high risk of bias. Given the nature of the mHealth intervention, participants and personnel were blinded in only 4 (17%) of the 23 studies [37,39,46,47,55]. In 13 (57%) of the 23 studies [24,26,27,35-37,40,44-48,55], the outcome assessors were blinded. Of the 23 studies, 2 (9%) were grade *A* (low bias), and 21 (91%) were grade *B* (moderate bias; Figure 2; Figure S1 in Multimedia Appendix 2) [24-27,35-53].

### Analysis of Overall Effects

#### QoL Measurement

**Figure 3:**
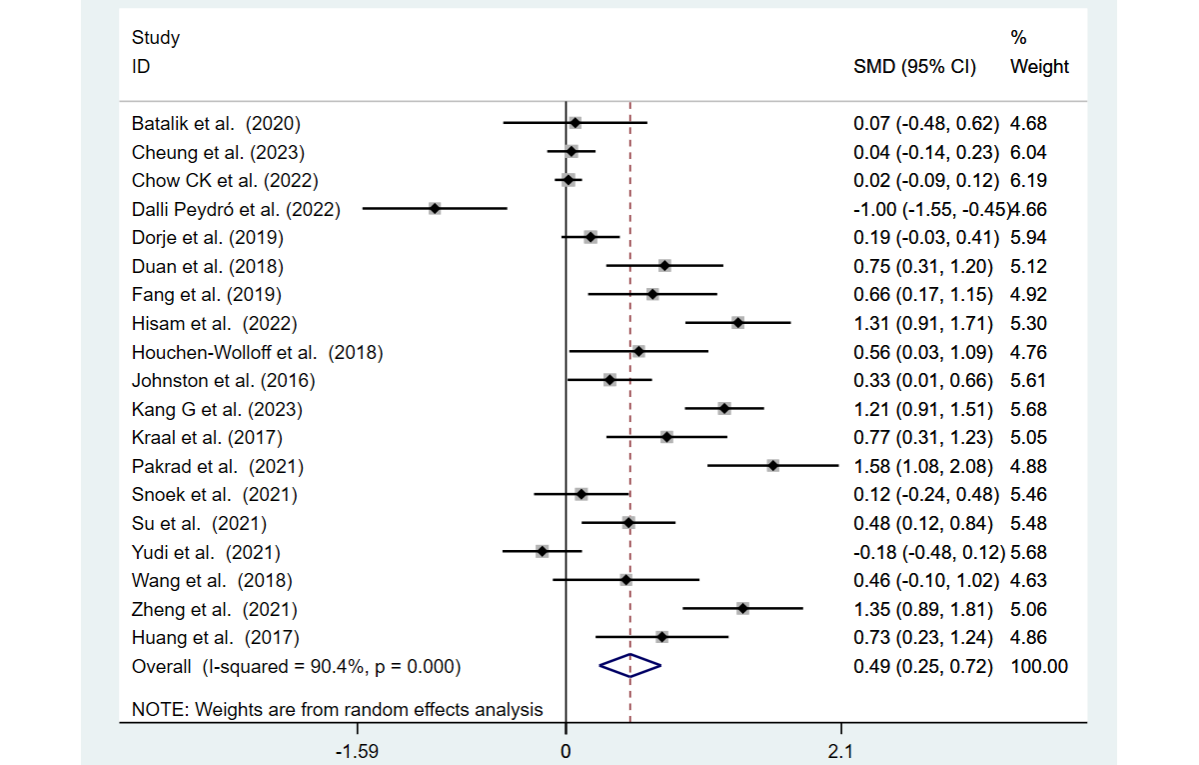
Forest plot of quality of life measured in 19 studies.

QoL was measured in 19 (83%) of the 23 studies. Figure 3 [24-27,35-42,44,45,47-49,51,53] displays the QoL scores from these 19 studies, illustrating that mHealth interventions significantly enhance QoL in patients with CHD compared to usual care (SMD 0.49, 95% CI 0.25-0.72; Z=4.07; *P*<.001). These studies showed considerable heterogeneity (*I*^2^=90.4%; *P*<.001).

#### Anxiety

**Figure 4:**
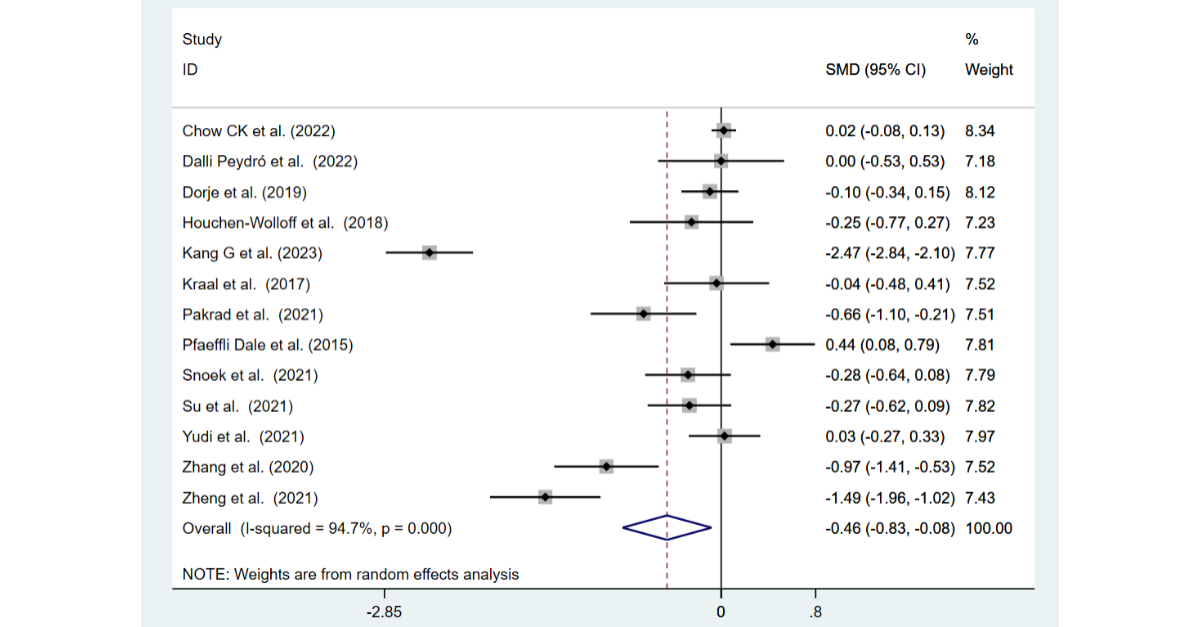
Forest plot of anxiety measured in 13 studies.

Anxiety was measured in 13 (57%) of the 23 studies. The anxiety scores displayed in Figure 4 [24-27,36,37,44,45,47,48,50-52] show that the mHealth interventions in these 13 studies relieved anxiety symptoms in patients with CHD compared to usual care (SMD −0.46, 95% CI −0.83 to −0.08; Z=2.38; *P*=.02). These studies revealed considerable heterogeneity (*I*^2^=94.7%; *P*<.001).

#### Depression

**Figure 5:**
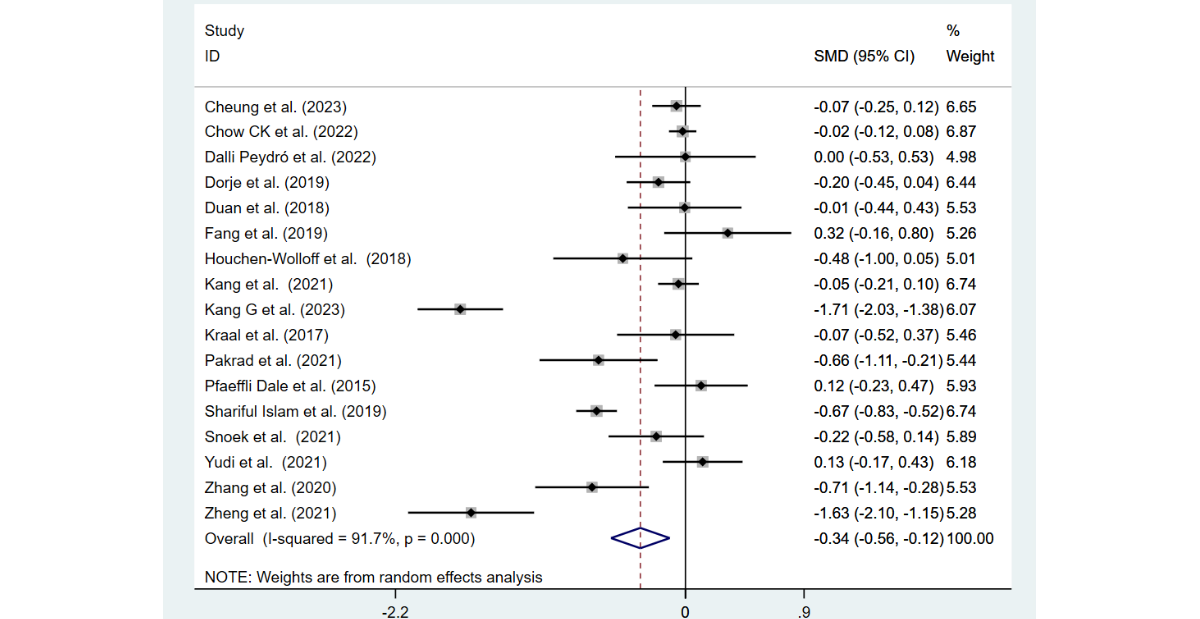
Forest plot of depression measured in 17 studies.

Depression was measured in 17 (74%) of the 23 studies. The depression scores displayed in Figure 5 [24-27,36-39,43-47,50-53] show that the mHealth intervention in these 17 studies relieved depressive symptoms in patients with CHD compared to usual care (SMD −0.34, 95% CI −0.56 to −0.12; Z=3.00; *P*=.003). These studies showed considerable heterogeneity (*I*^2^=91.7%; *P*<.001).

### Publication Bias

The funnel plots for QoL (Egger test: *P*=.02; Begg test: *P*=.58) indicated that there is a certain publication bias. However, the trim-and-fill plots showed that the results were robust before (*P*<.001) and after (*P*<.001) the trim-and-fill analysis. That is, there is a publication bias regarding QoL, but it does not affect the validity of the results (Figures S2 and S3 in Multimedia Appendix 2).

The funnel plots for anxiety (Egger test: *P*=.12; Begg test: *P*=.13) and depression (Egger test: *P*=.29; Begg test: *P*=.23) exhibited a generally symmetrical pattern. Furthermore, the trim-and-fill plots revealed that the effect sizes and *P* values did not vary before and after the trim-and-fill analysis, which was similar to the funnel plot results, showing that there was a low possibility of publication bias (Figures S4-S7 in Multimedia Appendix 2).

### Sensitivity Analysis

A sensitivity analysis was conducted to test whether the results may be significantly influenced by a single study by removing 1 study at a time and analyzing the remaining studies. The results showed that the overall effect was not considerably affected, indicating that the conclusions of this meta-analysis were relatively robust [34] (Figures S8-S10 in Multimedia Appendix 2).

### Subgroup Analysis

#### Different Durations of Interventions

##### The Effects on QoL

Both short-duration (<6 mo; SMD 0.60, 95% CI 0.21-0.98; *P*=.002; *I*^2^=83.8%) and long-duration (≥6 mo; SMD 0.42, 95% CI 0.11-0.72; *P*=.008; *I*^2^=92.5%) mHealth interventions were found to significantly enhance QoL among patients with CHD compared to usual care (Figure S11 in Multimedia Appendix 2).

##### The Effects on Anxiety and Depression

A significant effect favoring the mHealth intervention in mitigating anxiety (SMD −0.56, 95% CI −1.09 to −0.03; *P*=.04; *I*²=96.3%; Figure S12 in Multimedia Appendix 2) and depressive symptoms (SMD −0.45, 95% CI −0.73 to −0.18; *P*=.001; *I*²=93.8%; Figure S13 in Multimedia Appendix 2) compared to usual care was seen when only studies with long intervention durations (≥6 mo) were included, while this effect was not observed when only studies with short intervention durations (<6 mo) were included.

#### Different Intervention Methods: Simple Versus Complex Interventions

A significant effect favoring the mHealth intervention on enhancing QoL (SMD 0.57, 95% CI 0.28-0.86; *P*<.001; *I*²=92.1%; Figure S14 in Multimedia Appendix 2) as well as alleviating anxiety (SMD −0.73, 95% CI −1.29 to −0.16; *P*=.01; *I*²=96.7%; Figure S15 in Multimedia Appendix 2) and depressive symptoms (SMD −0.52, 95% CI −0.81 to −0.23; *P*<.001; *I*²=94.5%; Figure S16 in Multimedia Appendix 2) compared to usual care was seen when only studies using simple mHealth interventions were included, while this effect was not significant when only studies using complex mHealth interventions were included.

#### Influence of the COVID-19 Pandemic

##### The Effects on QoL

A comparative analysis of the studies conducted before the COVID-19 pandemic and those conducted during the pandemic revealed significant findings. The comparison of the studies conducted before the pandemic (14/19, 74%; SMD 0.30, 95% CI 0.14-0.45; *P*<.001; *I*^2^=70%) and those conducted during the pandemic (5/19, 26%; SMD 0.90, 95% CI 0.15-1.65; *P*=.02; *I*^2^=93.5%) indicated that mHealth interventions significantly enhanced QoL in patients with CHD compared to usual care, irrespective of the COVID-19 pandemic (Figure S17 in Multimedia Appendix 2).

##### The Effects on Anxiety and Depression

A significant effect favoring the mHealth intervention on reducing anxiety (SMD −1.16, 95% CI −2.24 to −0.08; *P*=.04; *I*^2^=95.7%; Figure S18 in Multimedia Appendix 2) and depressive symptoms (SMD −1.01, 95% CI −1.78 to −0.24; *P*=.01; *I*^2^=92%; Figure S19 in Multimedia Appendix 2) compared to usual care was seen when only studies conducted during the COVID-19 pandemic were included, while this effect was not significant when only studies conducted before the pandemic were included.

#### Different Country Types: LMICs Versus HICs

The mHealth interventions implemented in LMICs demonstrated greater efficacy in improving QoL (SMD 0.87, 95% CI 0.54-1.19; *P*<.001; *I*²=85.3%; Figure S20 in Multimedia Appendix 2) as well as alleviating anxiety (SMD −0.99, 95% CI −1.75 to −0.22; *P*=.01; *I*²=96.1%; Figure S21 in Multimedia Appendix 2) and depressive symptoms (SMD −0.66, 95% CI −1.23 to −0.08; *P*=.02; *I*²=93.6%; Figure S22 in Multimedia Appendix 2) in patients with CHD compared to usual care, while the mHealth interventions implemented in HICs showed limited effectiveness in enhancing these outcomes.

#### Different Intervention Contents: Focus on Mental Health

The mHealth interventions that focused on mental health proved significantly more effective in improving QoL (SMD 0.84, 95% CI 0.39-1.28; *P*<.001; *I^2^*=94.9%; Figure S23 in Multimedia Appendix 2) as well as reducing anxiety (SMD −0.84, 95% CI −1.47 to −0.20; *P*=.01; *I*^2^=97.1%; Figure S24 in Multimedia Appendix 2) and depression (SMD −0.81, 95% CI −1.40 to −0.22; *P*=.007; *I*^2^=96.3%; Figure S25 in Multimedia Appendix 2) in patients with CHD compared to usual care. However, there was no significant difference between mHealth interventions that were less relevant to mental health and usual care in improving QoL, anxiety, and depression outcomes in patients with CHD.

#### Different Levels of Adherence

##### The Effects on QoL

As depicted in Figure S26 in Multimedia Appendix 2, of the 19 studies, 10 (53%) were included in the lower adherence rates (<90%) group (SMD 0.44, 95% CI 0.16-0.72; *P*=.002; *I*^2^=83.4%), and 9 (47%) were included in the higher adherence rates (≥90%) group (SMD 0.53, 95% CI 0.10-0.97; *P*=.01; *I*^2^=94%). The findings indicated that mHealth interventions significantly enhanced QoL in patients with CHD, regardless of their level of adherence.

##### The Effects on Anxiety and Depression

Higher adherence rates (≥90%) were associated with superior outcomes in reducing anxiety (SMD −0.63, 95% CI −1.19 to −0.06; *P*=.03; *I*^2^=96.4%; Figure S27 in Multimedia Appendix 2) and depression (SMD −0.68, 95% CI −1.10 to −0.26; *P*=.002; *I*^2^=94.5%; Figure S28 in Multimedia Appendix 2) compared to usual care, while this effect was not observed when considering studies with lower adherence rates (<90%) alone.

## Discussion

### Principal Findings

We performed a meta-analysis of 23 RCTs (with 5406 participants in total) identified after a comprehensive database search to explore the effectiveness of mHealth interventions on QoL, anxiety, and depression in patients with CHD. Our meta-analysis revealed that mHealth interventions significantly enhanced QoL as well as reduced anxiety and depression in patients with CHD undergoing CR.

QoL is a comprehensive measurement index that encompasses various aspects of patient well-being, including physical, psychological, and social factors [56]. The relevant studies showed that there is a correlation between QoL, anxiety, and depression and other emotions of patients with CHD [57]. Furthermore, the incidence of cardiovascular disease has gradually increased in recent years, with high disability and mortality rates, making it the world’s leading health care burden and a major contributor to reduced QoL [58]. In our meta-analysis, mHealth interventions significantly enhanced QoL in patients with CHD compared to usual care. The reason may be that the mHealth interventions could provide patients with long-term and personalized health management knowledge, effectively increasing their awareness of the disease, improving their ability to self-manage, and promoting health behavior change [52] (eg, increasing physical activity [59], consuming a healthy diet, quitting smoking [60,61], and maintaining drug adherence [42]), thus improving cardiovascular health and QoL in patients with CHD [62].

In our analysis, we found that the mHealth interventions were effective in relieving anxiety and depressive symptoms in patients with CHD compared to usual care. Epidemiological studies have revealed a bidirectional link between anxiety, depression, and CHD. Anxiety and depression may induce coronary artery restenosis and cause an increase in the number of adverse cardiovascular events in patients with CHD [63]. At the same time, chest pain, chest tightness, fatigue, palpitation, and other symptoms caused by myocardial ischemia make patients prone to anxiety and other psychological problems [64-67]. Thus, helping patients manage stress and psychosocial risk factors is crucial.

The increased effectiveness of mHealth interventions could stem from the fact that patients with anxiety or depression often tend to be withdrawn, exhibiting reluctance to engage with others and express their emotions, and mHealth, as an accessible strategy, may be more conducive to enabling them to talk about their emotions and, as a consequence, alleviate their symptoms. In addition, compared to traditional intervention approaches, mHealth interventions are more convenient, provide timely feedback on patient issues, address patient distress, and improve communication between medical staff and patients so that patients can still receive systematic and continuous care outside the hospital [21]. In the study by Ivanova et al [68], the authors found that interventions delivered via the internet and smartphone apps were effective in reducing the anxiety of participants. Firth et al [69] found that anxiety could be decreased by using smartphone-delivered psychological interventions. Deady et al [70] indicated that a smartphone app could alleviate the symptoms of depression and possibly even avoid cases of incident depression.

Although this meta-analysis observed that mHealth interventions improved QoL as well as reduced anxiety and depression in patients with CHD, considerable heterogeneity remains. Therefore, we conducted subgroup analyses based on the different intervention durations, different intervention methods, the influence of the COVID-19 pandemic, different country types, different intervention contents, and different mHealth intervention adherence rates.

Regarding intervention durations, both intervention duration <6 months and intervention duration ≥6 months substantially enhanced QoL in patients with CHD, but intervention duration ≥6 months was superior to intervention duration <6 months in reducing anxiety and depression in patients with CHD. The reason may be that patients are unfamiliar with mHealth apps or wearable devices when they first start using an mHealth intervention, which may decrease the stickiness of mHealth use and the effect of the intervention. However, with the elapse of intervention time, patients gradually become well-versed in mHealth, realize the convenience and benefits of mHealth, and their compliance improves. Therefore, future research should concentrate on the long-term efficacy of mHealth, emphasizing health behavior maintenance and psychological outcomes.

In addition, a subgroup analysis revealed that a simple mHealth intervention is more effective than a complex mHealth intervention in promoting QoL and reducing anxiety and depression. There might be 2 reasons for this. First, CHD often affects middle-aged and older adults [71]. Older people have decreased cognitive function and memory, less access to the internet and mobile devices, and limited online exposure, and according to a prior study, older persons may have challenges accepting and adapting to complex informatization programs because they are from a different technological generation [72]. Second, complex interventions refer to the use of ≥2 mHealth methods, which are more difficult to use than simple interventions and may decrease the stickiness of mHealth use. Therefore, the application of mHealth interventions should consider the receptivity and memory of middle-aged and older adults, enhance the development of mHealth software and hardware, increase the use of images and videos, enlarge the font size of pages, consider the individual needs of patients, and motivate patients in an accessible and understandable way to actively participate in the mHealth program [73].

Furthermore, a subgroup analysis was performed according to whether the study was conducted before or during the COVID-19 pandemic and found that, during the pandemic, the use of mHealth was more conducive to improving anxiety and depressive symptoms in patients with CHD. The reasons for this are as follows. First, several surveys conducted during the COVID-19 pandemic showed a higher prevalence of anxiety and depression as well as lower well-being compared to previous estimates [74,75]. Second, the COVID-19 pandemic exerted great pressure on medical resources, and some medical institutions needed to prioritize the treatment of patients with COVID-19 infection, leaving other patients’ medical needs unmet. mHealth technology can help patients with CHD to obtain timely medical services and psychological support and reduce anxiety and depression caused by the strain on medical resources [76]. Third, the COVID-19 pandemic has heightened people’s awareness of health, and patients are more willing to self-manage and monitor their health using mHealth technology to minimize outdoor exposure. Through self-management, patients can better manage their condition and reduce anxiety and depression. Finally, during the COVID-19 pandemic, the use of mHealth increased, with a wider variety of applications offering more features that better met the personalized needs of patients and helped them relieve anxiety and depression.

Regarding different country types, the results showed that the use of mHealth was more conducive to improving QoL in patients with CHD in LMICs and reducing their anxiety and depressive symptoms. The reason may be that health care resources in LMICs are often scarcer than in HICs, making it difficult for patients to access timely medical care and mental health support. mHealth technology can compensate for the lack of health care resources by allowing patients to access medical information and mental health support through mobile phones or other mobile devices. In addition, mHealth technology can reduce the cost of health care services because patients do not have to pay for extra transportation and do not need to spend time waiting in line. This is particularly important for patients with CHD in LMICs because they often make up a group that is considered economically disadvantaged.

On the basis of our analysis, there are 2 possible reasons why mHealth interventions that focus on mental health could improve QoL and relieve anxiety and depressive symptoms in patients with CHD. First, mHealth interventions focusing on mental health can provide mental health education, psychological support, and other services, which can help relieve psychological problems such as anxiety and depression, thereby improving QoL in patients. Second, mHealth interventions focusing on mental health can help patients better self-monitor and self-manage, including self-monitoring physiological indicators and recording health behavior. This helps patients to better grasp their health status, improve their QoL, and relieve anxiety and depression.

Regarding different levels of adherence, we found that mHealth was more effective in relieving anxiety and depression in patients with CHD with higher adherence rates (≥90%) than those with lower adherence rates (<90%). There are several possible reasons why the adherence rates differed. First, as shown in our results, lower adherence may be caused by low engagement with the mHealth intervention, which is normal and in line with other research on health behavior change [77,78]. Thus, it is strongly advised to enhance the functionality of mHealth tools, such as apps and wearables, to increase adherence to mHealth interventions. This enhancement should focus on meeting the individual needs of patients with CHD and supporting their engagement. Su et al [79] underscore the significance of incorporating patients’ preferences in the design of mHealth platforms and highlight the essential role of features such as self-monitoring and self-evaluation in improving user engagement and ensuring the effective use of these tools in real-world settings. Second, financial rewards may increase patient enthusiasm to complete interventions. Therefore, the incentive mechanism is indispensable in the intervention process. In addition, the mHealth intervention duration, intervention frequency, and intervention methods may also affect the compliance of patients.

Safety is a priority for all treatments. Despite the promising results of this study, there are general concerns about patient safety in unsupervised mHealth interventions, particularly with regard to physical activity. Notably, of the 23 RCTs, 12 (52%) addressed adverse safety events, and all 12 reported no intervention-related adverse events or deaths, suggesting a certain level of safety in mHealth interventions. In addition, a systematic review assessed the safety of home-based CR (HBCR) programs (digital or telehealth interventions) and concluded that the risk of adverse events occurring is low, thus reassuring the safety of such interventions [80]. Another review examined the incidence and severity of adverse events associated with HBCR and revealed a low rate of major exercise-related adverse events [81]. Furthermore, a systematic review that explored and evaluated the effectiveness of technology-assisted CR demonstrated that digital therapeutics–based CR is not only safe and feasible for patients with CHD but also enjoys a high level of satisfaction among patients [82].

In terms of the cost-effectiveness of mHealth, several reviews have indicated that mHealth interventions exhibit promising or comparable cost-effectiveness when compared to usual care [80,83-85]. A review conducted by McDonagh et al [80] concluded that the cost per patient for center-based CR and HBCR programs (digital or telehealth interventions) was similar. Another review highlighted the cost-effectiveness of exercise-based telehealth CR and recommended its use to reduce the economic burden of preventive health care and enhance CR accessibility [83]. In addition, Brouwers et al [86] evaluated the cost-effectiveness of cardiac telerehabilitation with relapse prevention compared with center-based CR among patients with coronary artery disease and indicated that, from a societal perspective, telerehabilitation was associated with lower costs compared with center-based CR (mean US $23,405, SE US $3142 vs mean US $27,843, SE US $4126, respectively).

There are 3 factors contributing to the cost-effectiveness of mHealth interventions. First, as demonstrated in an RCT, mHealth interventions could facilitate more frequent patient-specialist interactions, fostering enhanced interpersonal engagement that could increase patient participation and self-efficacy in health management [87]. Second, mHealth interventions could offer responsive, personalized services, leading to increased patient adherence to CR protocols and enhanced rehabilitation outcomes. Third and last, mHealth has the potential to enhance medical service coverage, particularly benefiting individuals with limited access to CR, such as those residing in remote or rural areas.

### Comparison With Prior Work

According to our literature review, there is a substantial body of research exploring the application of mHealth. However, to the best of our knowledge, no comprehensive study has been conducted on the impact of mHealth on QoL, anxiety, and depression outcomes in patients with CHD.

Among the published studies, Rintala et al [88] reviewed 11 studies and showed that mHealth applications are promising for enhancing QoL in stroke rehabilitation. Chin-Jung et al [89] found that mHealth interventions significantly improved life satisfaction and mental health in patients with type 1 diabetes. Dawes et al [90] indicated that postsurgery QoL improvements were expedited by mHealth. Ni et al [91] performed a comprehensive review of 19 RCTs, and the findings revealed that while mHealth reduced post–cardiac surgery depression and improved the physical aspect of QoL, it had no impact on the psychological aspect of QoL and anxiety in patients after cardiac surgery (subgroup analyses of the main outcomes were not performed).

Compared to previous studies, our study focused on QoL, anxiety, and depression in patients with CHD; we performed subgroup analyses based on the different intervention durations, different intervention methods, the influence of the COVID-19 pandemic, different country types, different intervention contents, and different levels of adherence to detect the source of heterogeneity; and we included a larger number of RCTs. Moreover, we followed the PRISMA guidelines for this meta-analysis to ensure that our methodology was robust and reliable [92].

### Limitations

While we have attempted to perform a comprehensive meta-analysis of the effects of mHealth on QoL, anxiety, and depression in patients with CHD, some limitations need to be considered. First, some studies have methodological flaws (eg, allocation sequence concealment and a lack of blinding) that may influence the accuracy of this meta-analysis. Hence, the findings should be taken with caution, and future researchers are advised to carefully execute randomized controlled designs and provide more data following reporting criteria to ensure the study’s quality. Second, although we considered the COVID-19 pandemic an important confounding factor and conducted subgroup analyses to address the influence of the pandemic on studies, we did not include a postpandemic subgroup for analysis. That is because, based on our database search period for this meta-analysis, we excluded studies published after August 12, 2023, by which time the COVID-19 pandemic was no longer considered a major public health event, and no RCT studies meeting the inclusion and exclusion criteria of this study had been retrieved.

Third, although we carried out subgroup analyses to determine effects within categories, the social determinants of health such as education level, income status, access to mHealth technologies, marital status, and so on, were difficult to consider because most of the included studies (19/23, 83%) did not report detailed information on the social determinants of health. Fourth, although mHealth interventions were used in all intervention groups, the wide range of intervention types, from SMS text messaging and mobile apps to wearables, may significantly contribute to the diversity in outcomes among the studies. Furthermore, because many studies (7/23, 30%) used >1 mHealth intervention, we only conducted a subgroup analysis for simple versus complex mHealth interventions, rather than different types of mHealth interventions specifically. Fifth, most of the studies (20/23, 87%) included in our meta-analysis only reported that the enrolled patients were patients with CHD; the specific subtype of CHD was not clarified. Consequently, while our findings indicate a positive effect of mHealth interventions on QoL and mental health (anxiety and depression) in patients with CHD, we are unable to determine the effectiveness across different CHD subtypes conclusively. Sixth and last, the sample sizes varied greatly among the included studies. Further research should focus on increasing the sample sizes and strengthening the randomization, allocation, and blinding methodologies to improve the quality of evidence.

### Conclusions

mHealth interventions effectively improved QoL, anxiety, and depression in patients with CHD. Specifically, mHealth interventions effectively alleviated anxiety and depression among patients with CHD when (1) the intervention duration was ≥6 months, (2) the mHealth intervention was a simple one (only 1 mHealth intervention was used), (3) it was implemented during the COVID-19 pandemic, (4) it was implemented in LMICs, (5) the intervention focused on mental health, and (6) adherence rates were high (≥90%). Furthermore, a notable impact favoring mHealth interventions in enhancing QoL compared to usual care was evident in scenarios in which (1) the mHealth intervention was a simple one, (2) it was implemented in LMICs, and (3) the intervention focused on mental health. However, because most of the studies (21/23, 91%) we reviewed were of moderate quality, we must proceed with caution in applying the evidence. Large sample, high-quality, and rigorously designed RCTs are needed to provide more evidence.

## Appendix

Multimedia Appendix 1 PRISMA (Preferred Reporting Items for Systematic Reviews and Meta-Analyses) checklist.

Multimedia Appendix 2 Sensitivity analyses, subgroup analyses results, and the search strategies used for different databases.

## Abbreviations

CHD: coronary heart disease
CR: cardiac rehabilitation
HBCR: home-based cardiac rehabilitation
HIC: high-income country
LMIC: low- or middle-income country
mHealth: mobile health
PICOS: population, intervention, comparison, outcomes, and study design
PRISMA: Preferred Reporting Items for Systematic Reviews and Meta-Analyses
QoL: quality of life
RCT: randomized controlled trial
SMD: standardized mean difference
