# Supplementary material for: The Effects of mHealth Interventions on Quality of Life, Anxiety, and Depression in Patients With Coronary Heart Disease: Meta-Analysis of Randomized Controlled Trials
Source: J Med Internet Res. 2024 Jun 11;26:e52341. doi: 10.2196/52341 (PMC11200038; doi:10.2196/52341)
Supplement: Multimedia Appendix 2 [file jmir_v26i1e52341_app2.pdf]

Multimedia Appendix

Effects of mobile health interventions on quality of life, anxiety, and depression outcomes in patients with coronary heart disease: A Meta-analysis of randomized controlled trial

Table of contents

Figure S1. Risk of bias summary 1
Quality of life 2
Figure S2. Funnel plot of quality of life 2
Figure S3. Trim and fill plots of quality of life 2
Anxiety 3
Figure S4. Funnel plot of anxiety 3
Figure S5. Trim and fill plots of anxiety 3
Depression 4
Figure S6. Funnel plot of depression 4
Figure S7. Trim and fill plots of depression 4
Sensitivity analyses. 5
Figure S8. Sensitivity analysis of quality of life 5
Figure S9. Sensitivity analysis of anxiety 5
Figure S10. Sensitivity analysis of depression 6
Subgroup analyses: Results stratified by different durations of interventions (<6 months vs. ⩾6 months) 6
Figure S11. Mean effect sizes of quality of life 6
Figure S12. Mean effect sizes of anxiety 7
Figure S13. Mean effect sizes of depression 7
Subgroup analyses: Results stratified by different intervention methods (simple intervention vs. complex intervention) 8
Figure S14. Mean effect sizes of quality of life 8
Figure S15. Mean effect sizes of anxiety 8
Figure S16. Mean effect sizes of depression 9
Subgroup analyses: Results stratified by influence of the COVID-19 pandemic (before the pandemic vs. during the pandemic) 9
Figure S17. Mean effect sizes of quality of life 9
Figure S18. Mean effect sizes of anxiety 10
Figure S19. Mean effect sizes of depression 10

Subgroup analyses: Results stratified by different country types (LMICs vs. HICs) 11
Figure S20. Mean effect sizes of quality of life 11
Figure S21. Mean effect sizes of anxiety 11
Figure S22. Mean effect sizes of depression 12
Subgroup analyses: Results stratified by different intervention content (whether the mHealth intervention focused on mental health) 12
Figure S23. Mean effect sizes of quality of life 12
Figure S24. Mean effect sizes of anxiety 13
Figure S25. Mean effect sizes of depression 13
Subgroup analyses: Results stratified by different levels of adherence (higher adherence rates-⩾90% vs. lower adherence rates-<90%) 14
Figure S26. Mean effect sizes of quality of life 14
Figure S27. Mean effect sizes of anxiety 14
Figure S28. Mean effect sizes of depression 15
Searching strategies 16
Literature search in PubMed 16
Literature search in Embase 17
Literature search in Cochrane 18
Literature search in Web of Science 19
Literature search in CINAHL 20

Figure S1. Risk of bias summary

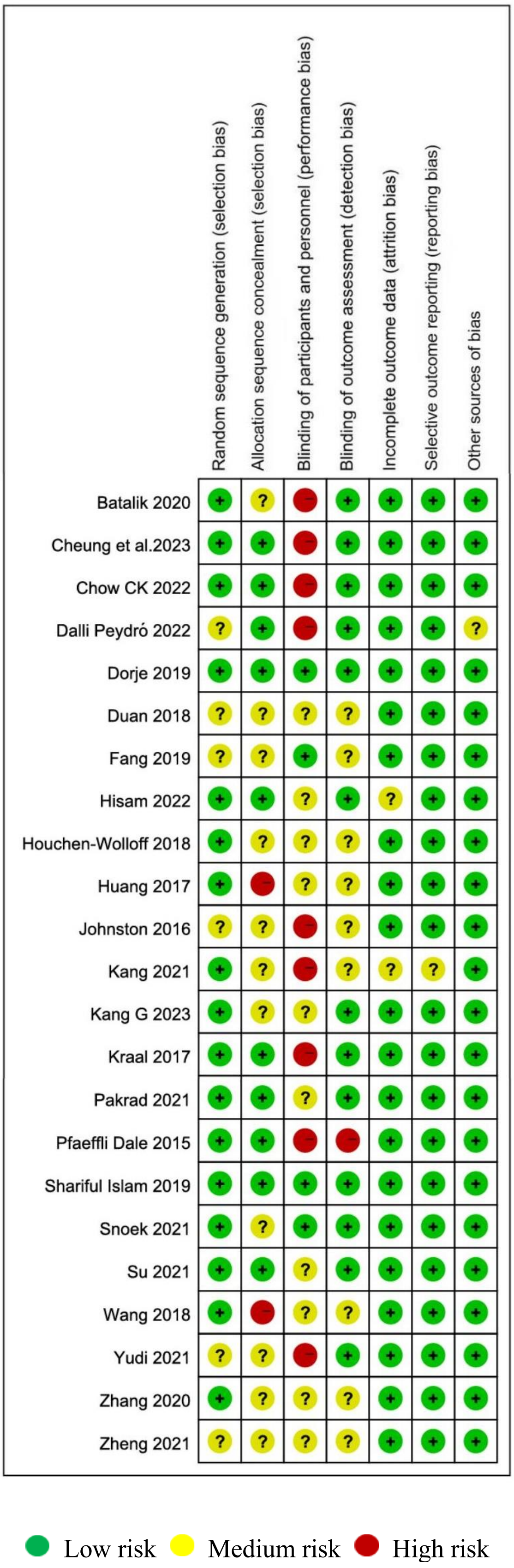

Quality of life

Figure S2. Funnel plot of quality of life

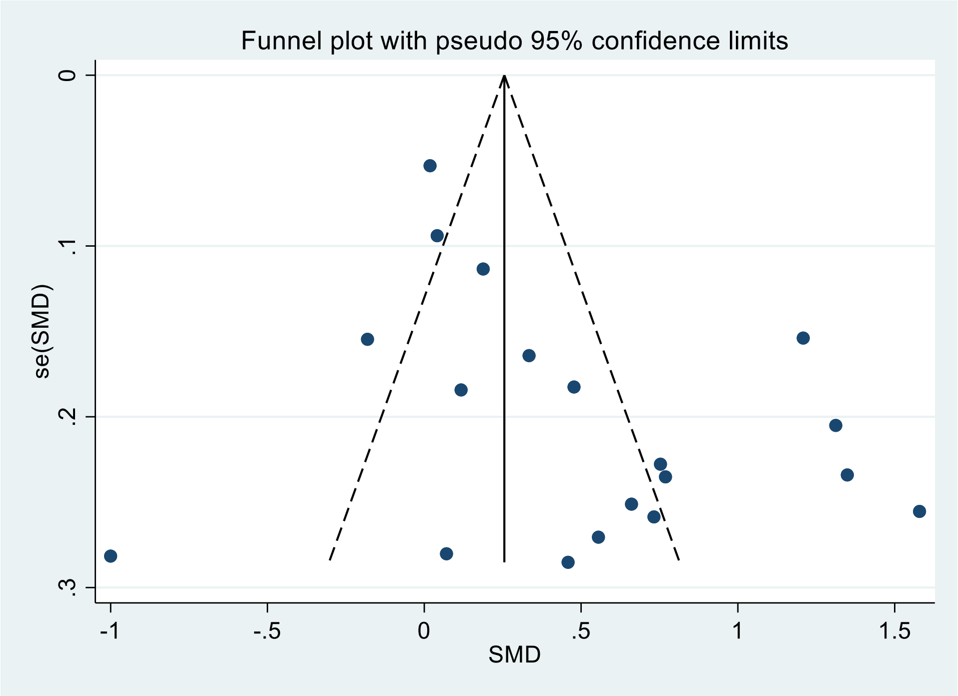

Figure S3. Trim and fill plots of quality of life

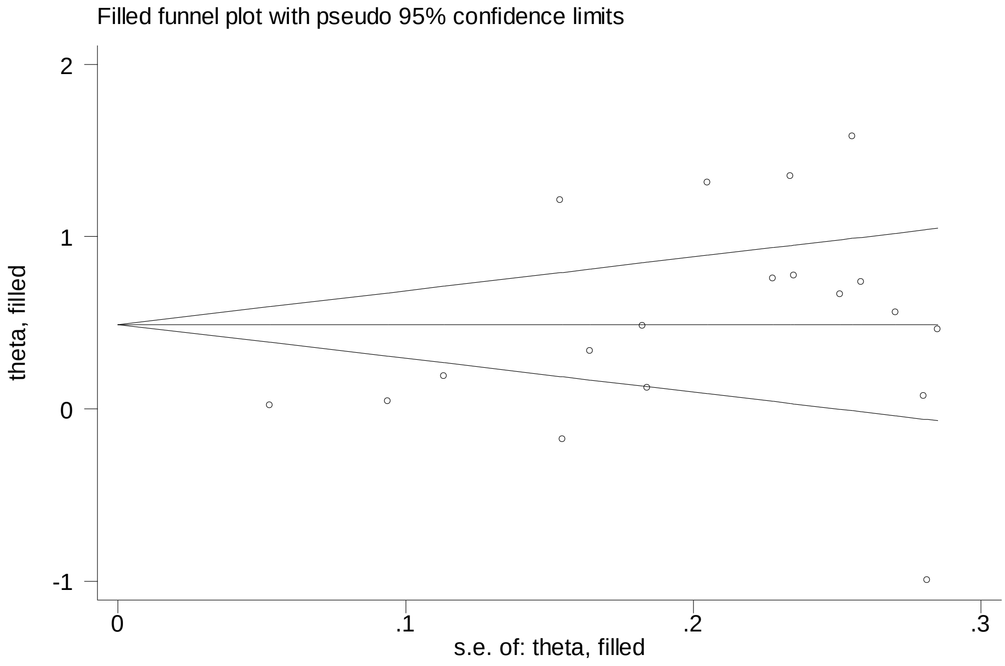

Based on 19 studies: 19 randomized trials and 0 imputed trials. The results were stable before (*P*<0.001) and after (*P*<0.001) the trim and fill analysis.

Anxiety

Figure S4. Funnel plot of anxiety

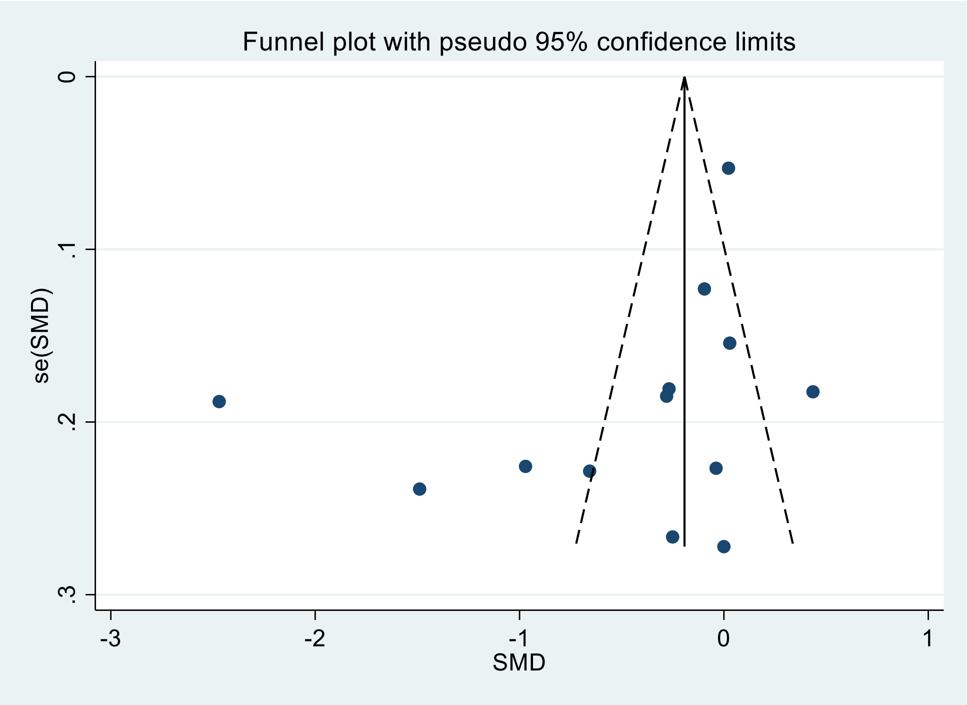

Figure S5. Trim and fill plots of anxiety

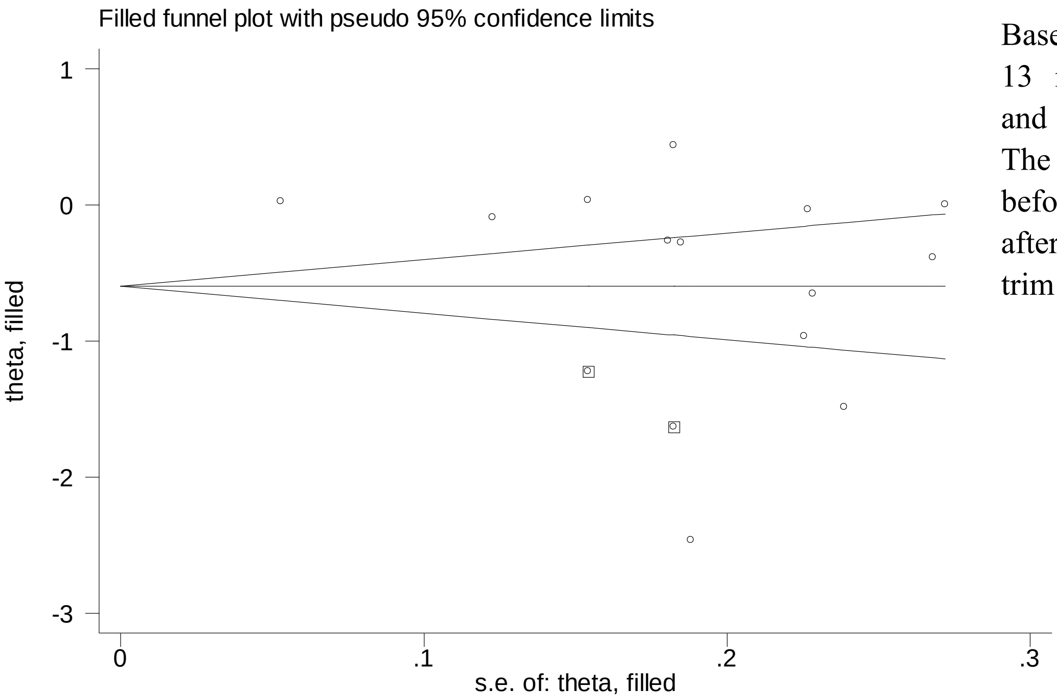

Based on 15 studies: 13 randomized trials and 2 imputed trials. The results were stable before (*P*=0.017) and after (*P*=0.003) the trim and fill analysis.

Depression

Figure S6. Funnel plot of depression

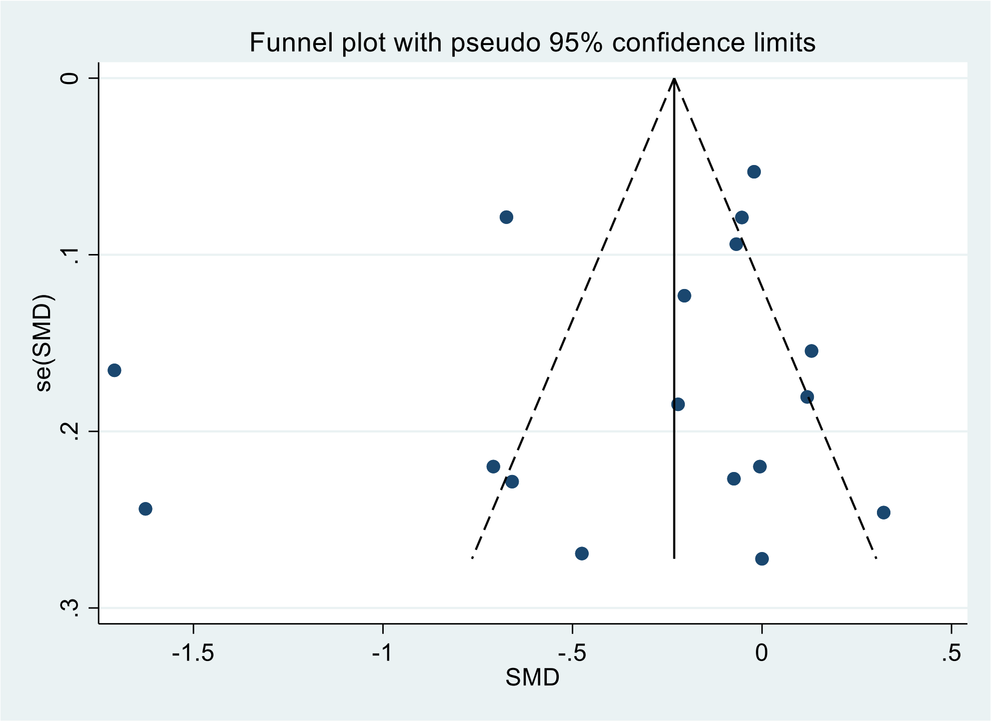

Figure S7. Trim and fill plots of depression

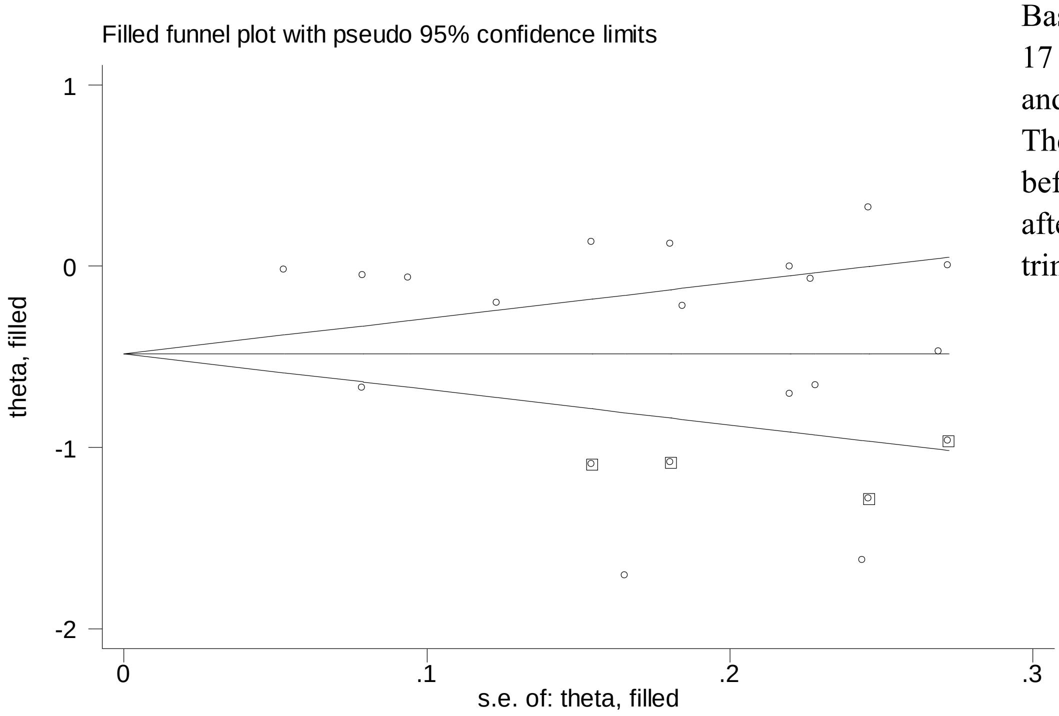

Based on 21 studies: 17 randomized trials and 4 imputed trials. The results were stable before (P=0.003) and after (P<0.001) the trim and fill analysis.

Sensitivity analyses.

Figure S8. Sensitivity analysis of quality of life

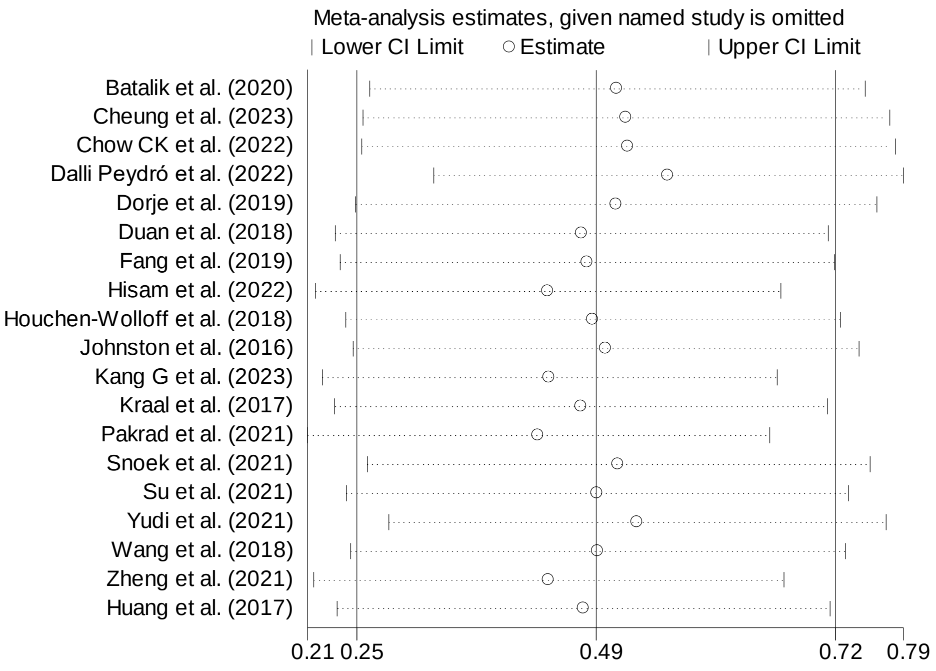

Figure S9. Sensitivity analysis of anxiety

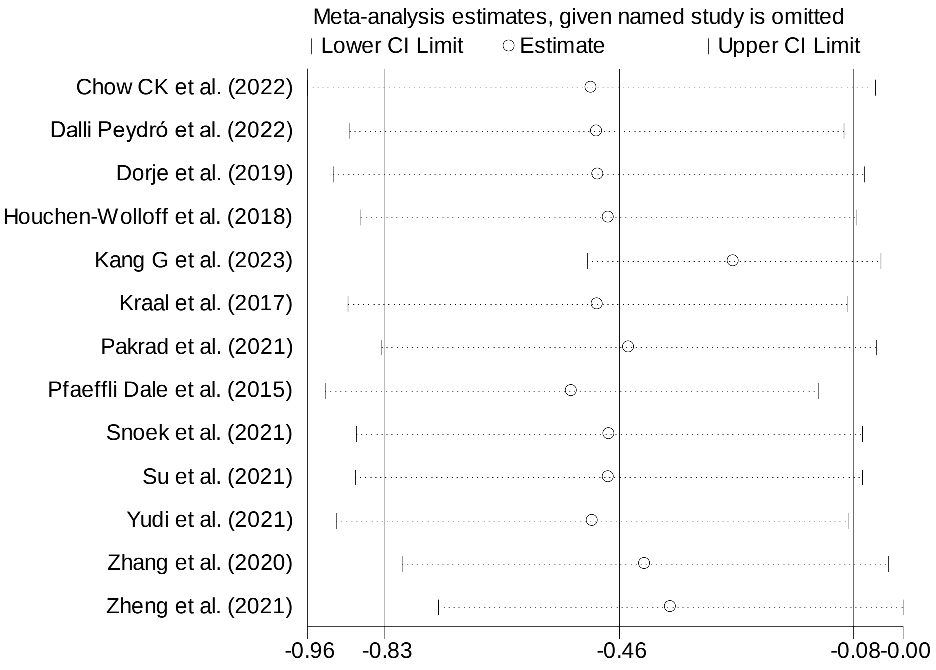

Figure S10. Sensitivity analysis of depression

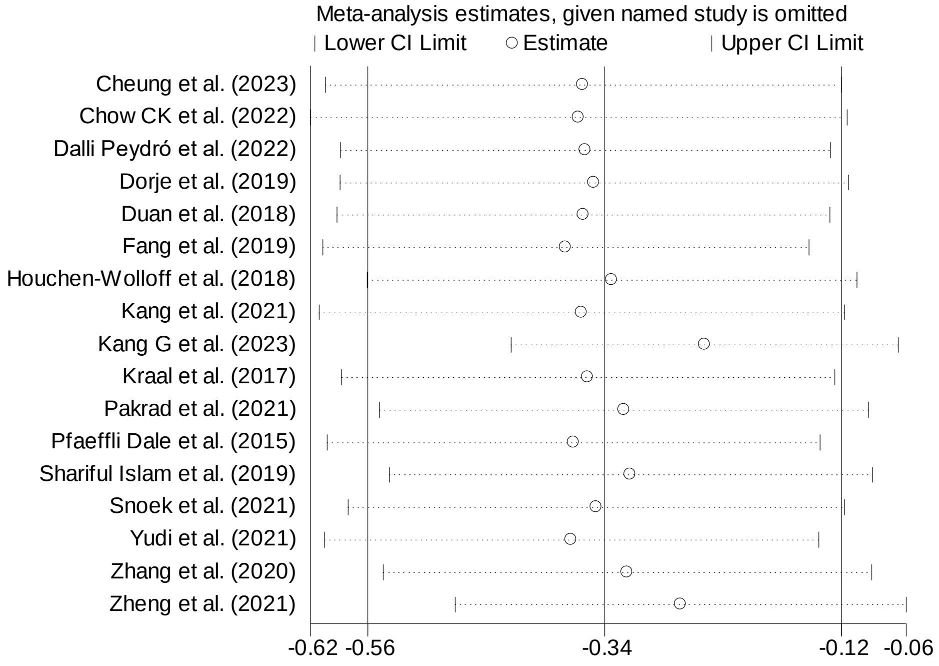

Subgroup analyses: Results stratified by different durations of interventions (< 6 months vs. ≥6 months)

Figure S11. Mean effect sizes of quality of life

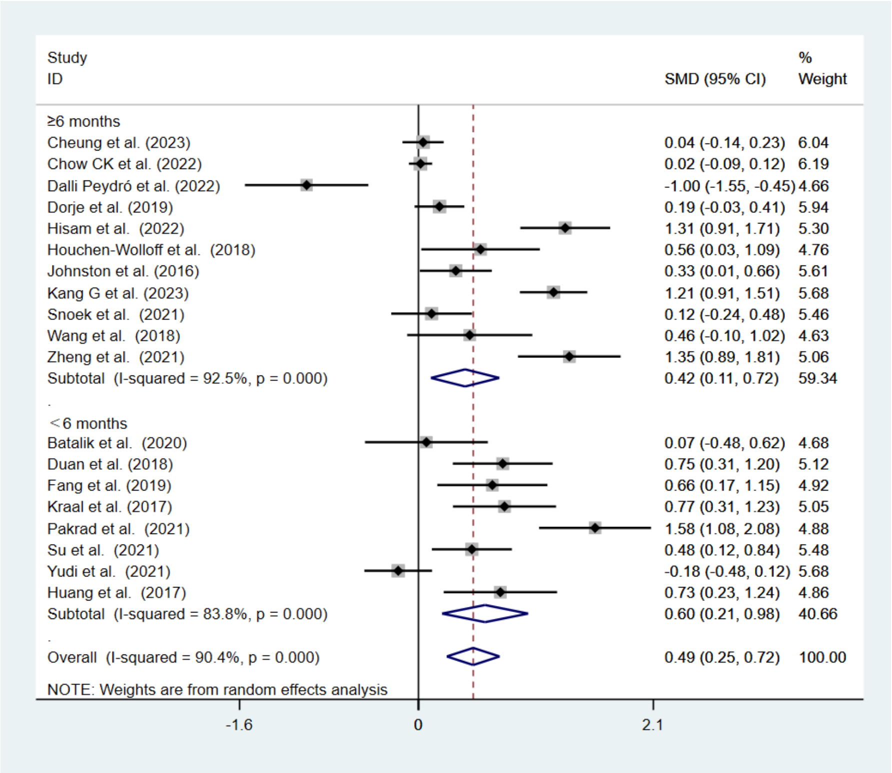

Figure S12. Mean effect sizes of anxiety

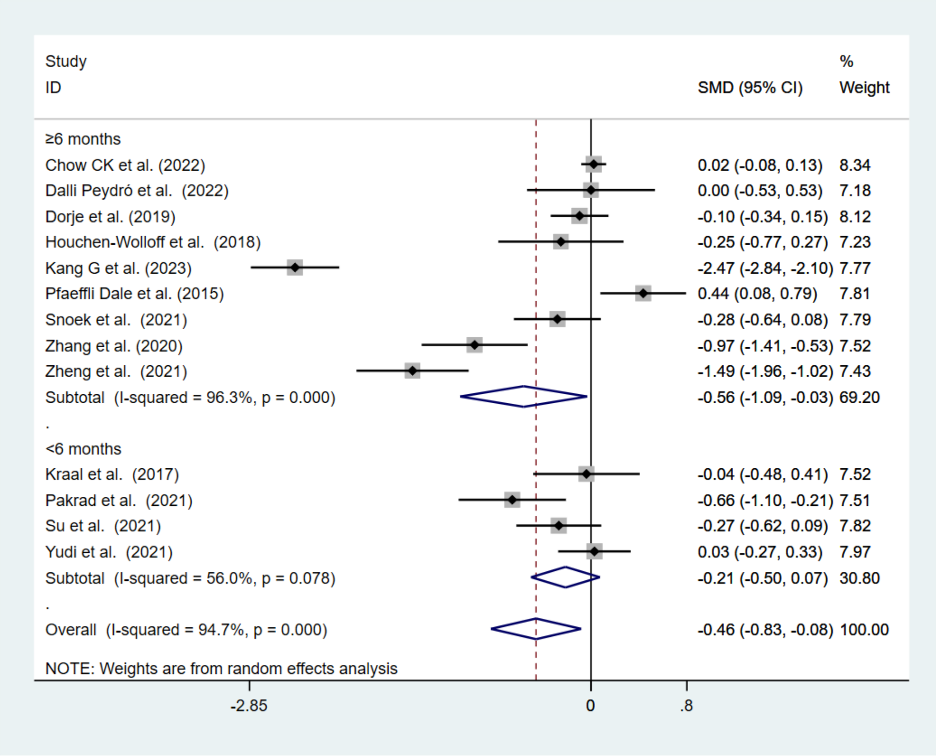

Figure S13. Mean effect sizes of depression

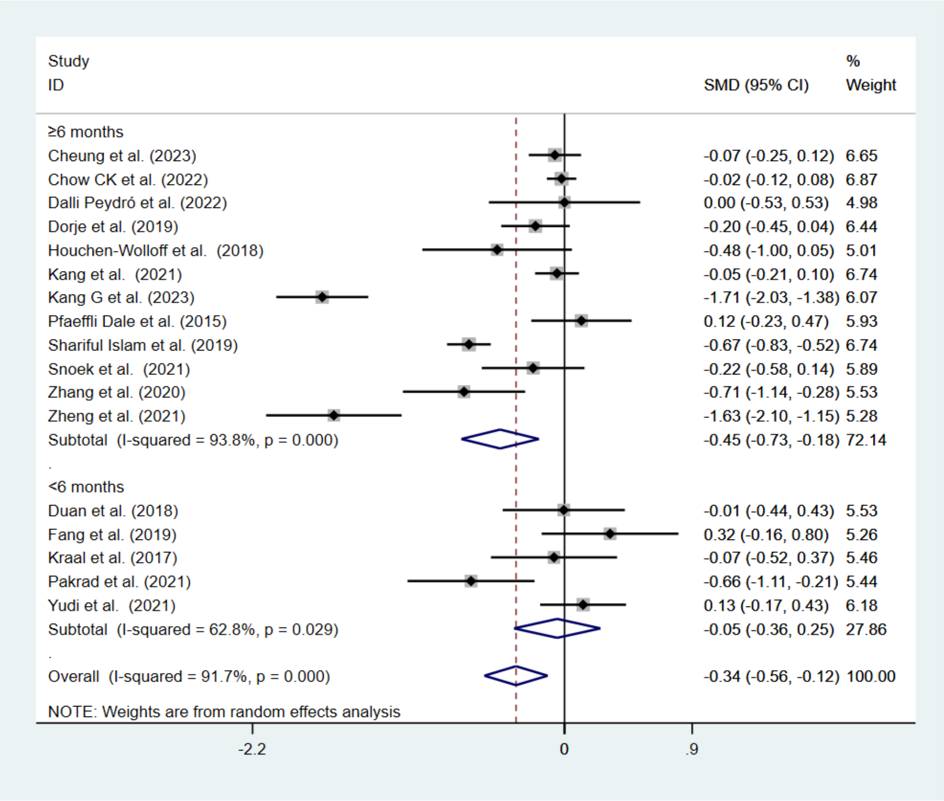

Subgroup analyses: Results stratified by different intervention methods (simple intervention vs. complex intervention)

Figure S14. Mean effect sizes of quality of life

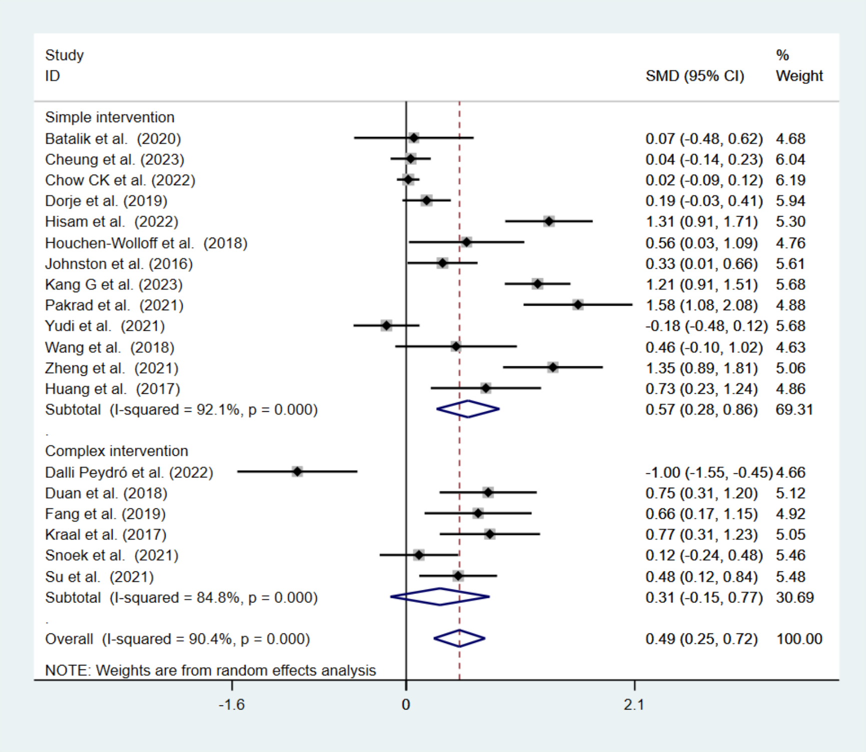

Figure S15. Mean effect sizes of anxiety

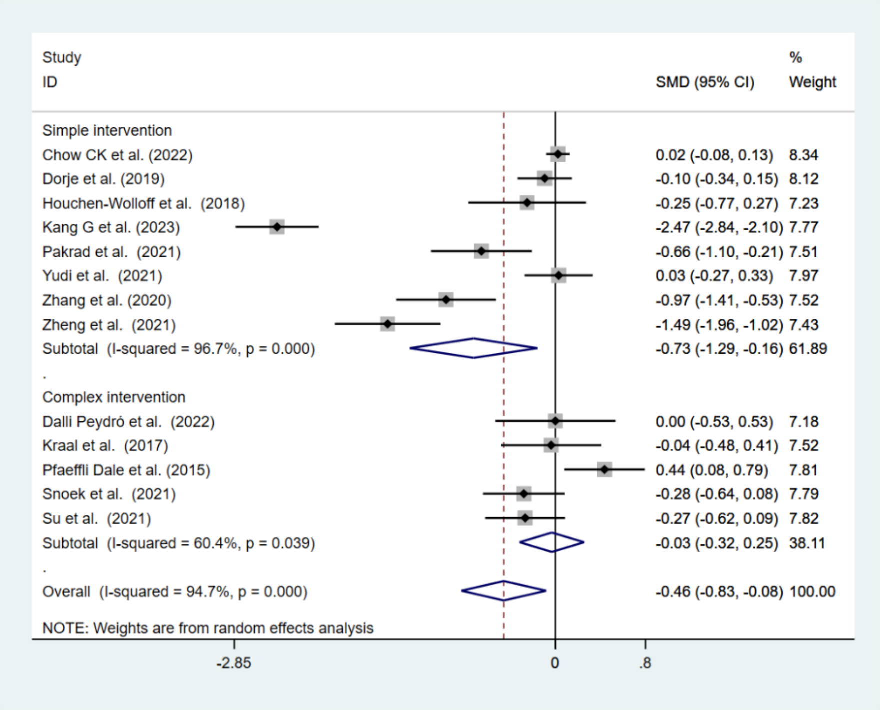

Figure S16. Mean effect sizes of depression

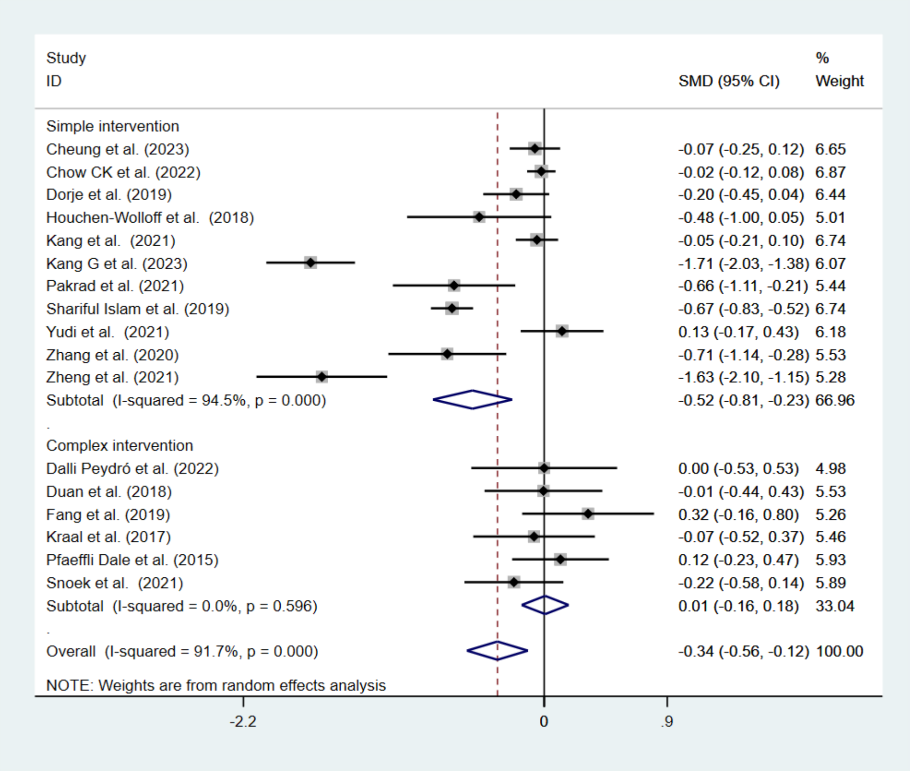

Subgroup analyses: Results stratified by influence of the COVID-19 pandemic (before the pandemic vs. during the pandemic)

Figure S17. Mean effect sizes of quality of life

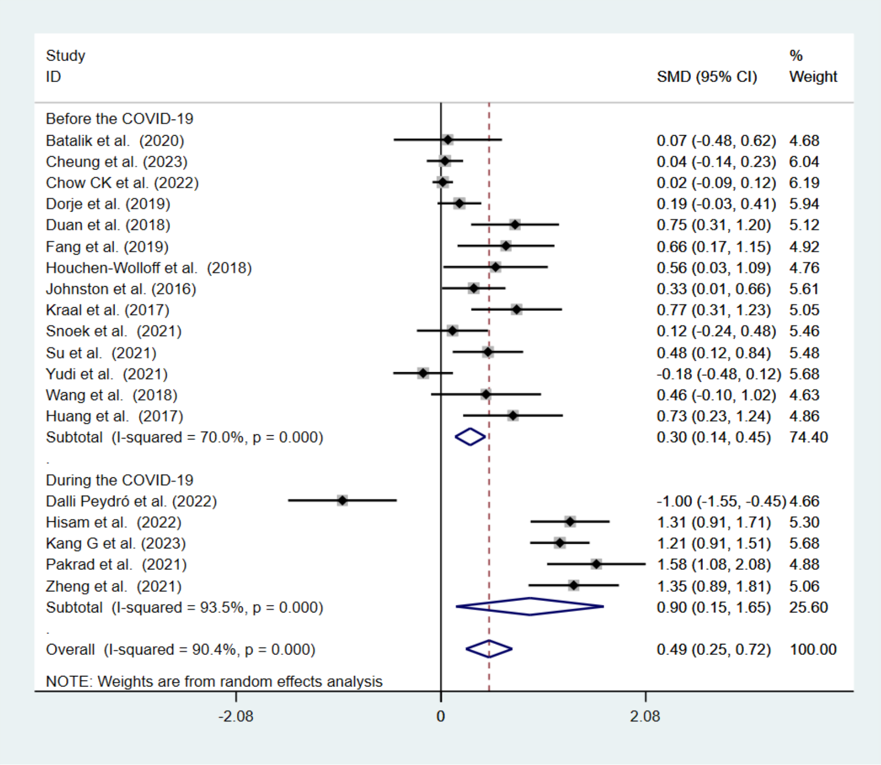

Figure S18. Mean effect sizes of anxiety

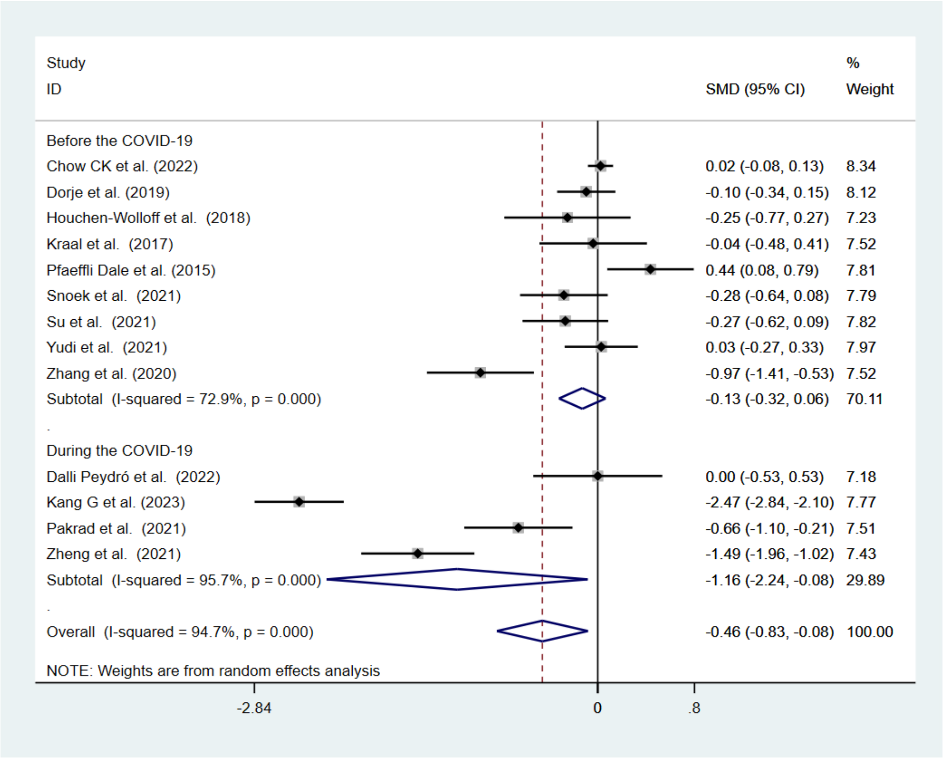

Figure S19. Mean effect sizes of depression

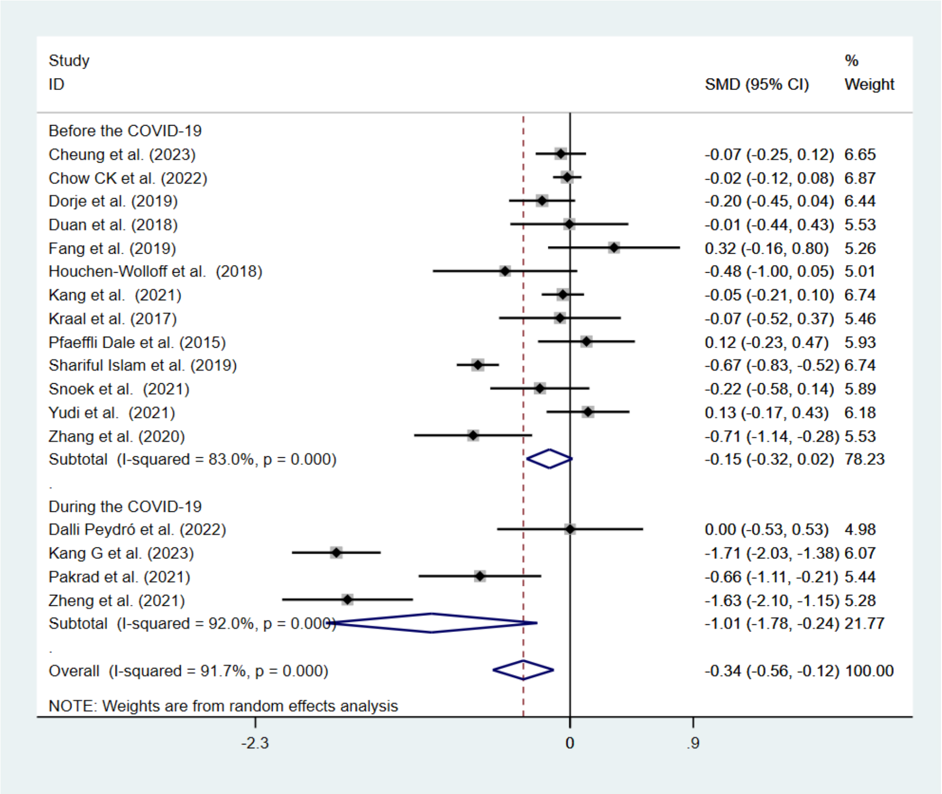

Subgroup analyses: Results stratified by different country types (LMICs vs. HICs)

Figure S20. Mean effect sizes of quality of life

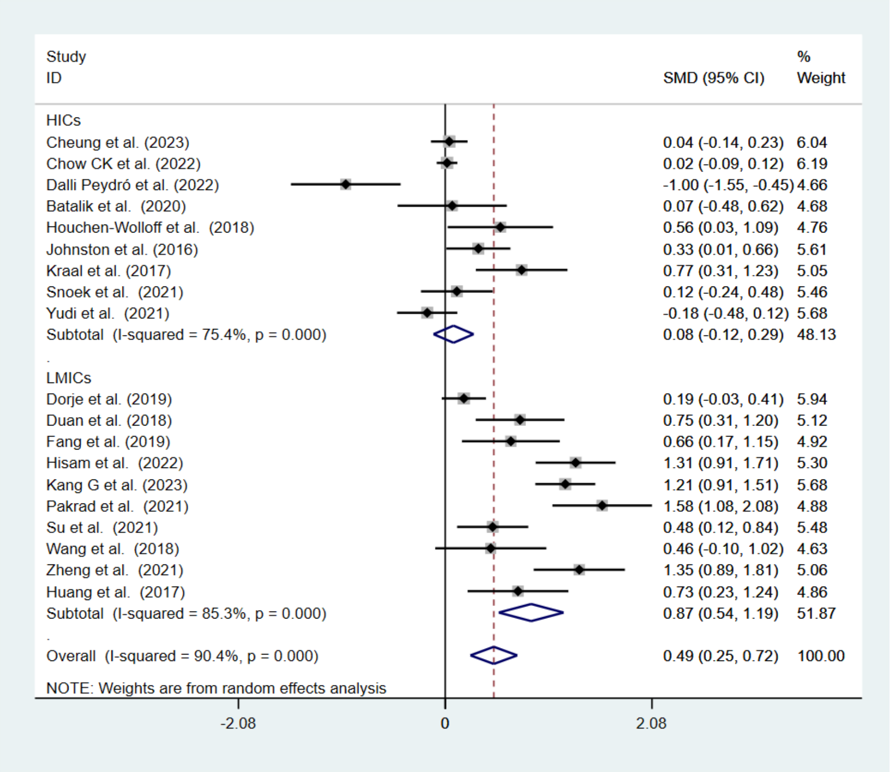

Figure S21. Mean effect sizes of anxiety

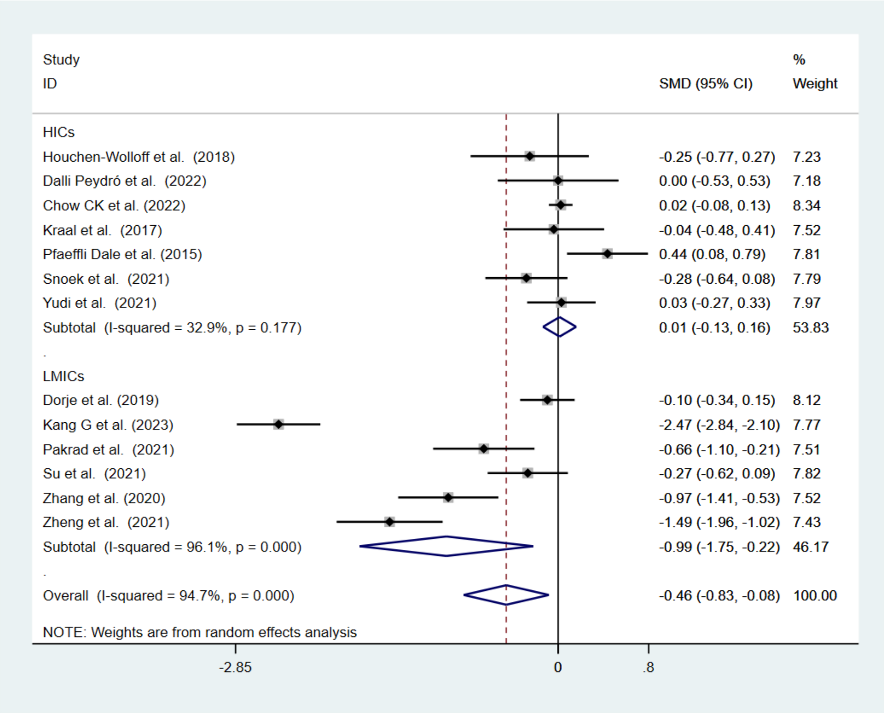

Figure S22. Mean effect sizes of depression

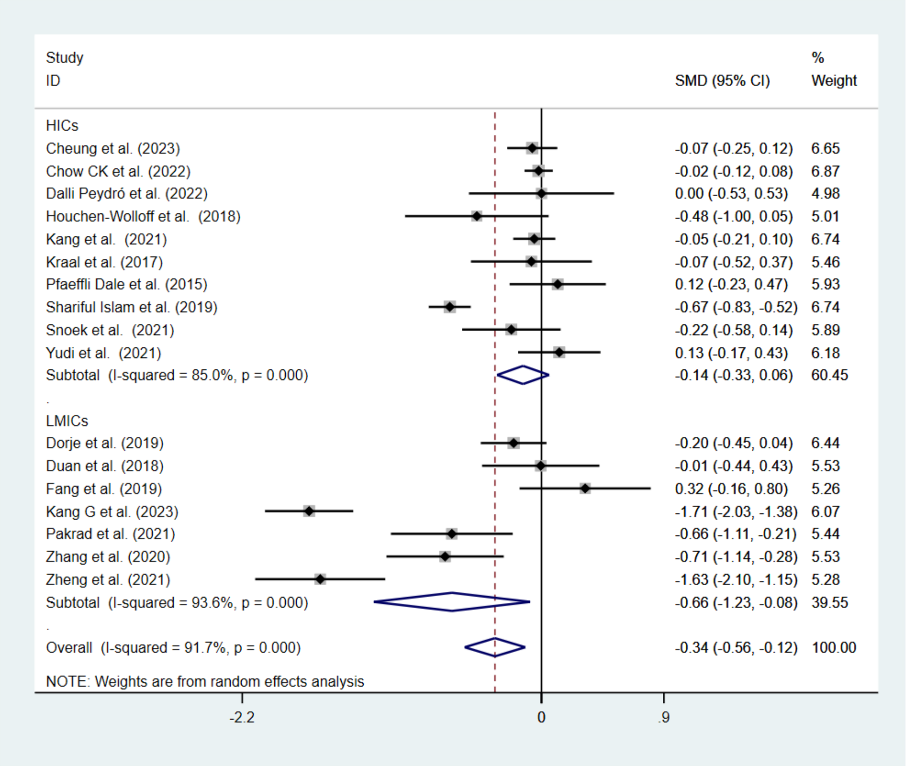

Subgroup analyses: Results stratified by different intervention content (whether the mHealth intervention focused on mental health)

Figure S23. Mean effect sizes of quality of life

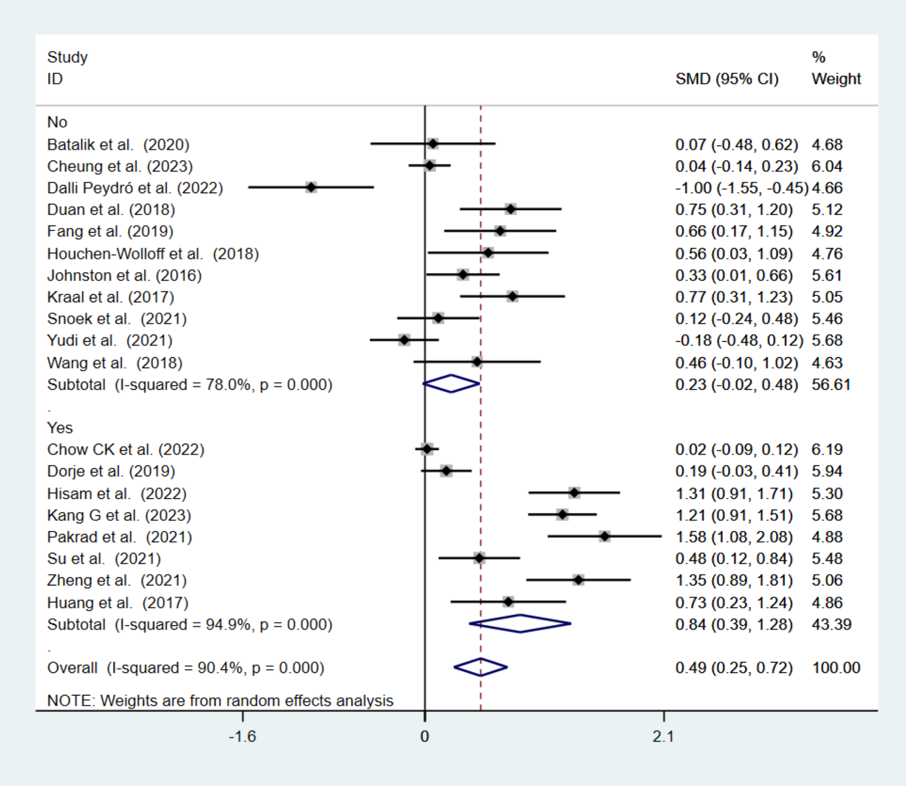

Figure S24. Mean effect sizes of anxiety

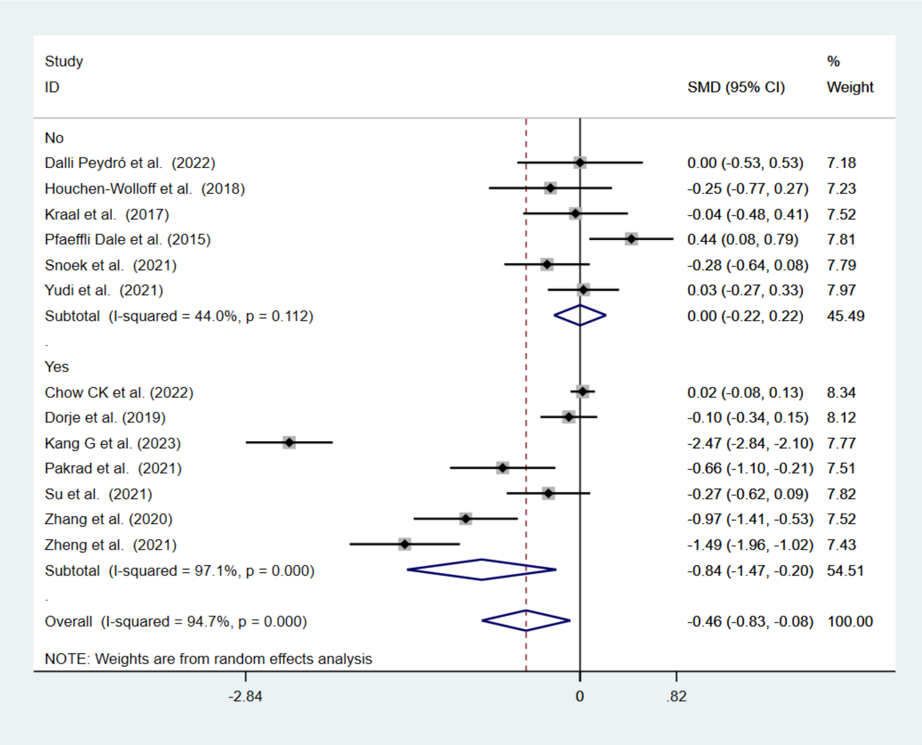

Figure S25. Mean effect sizes of depression

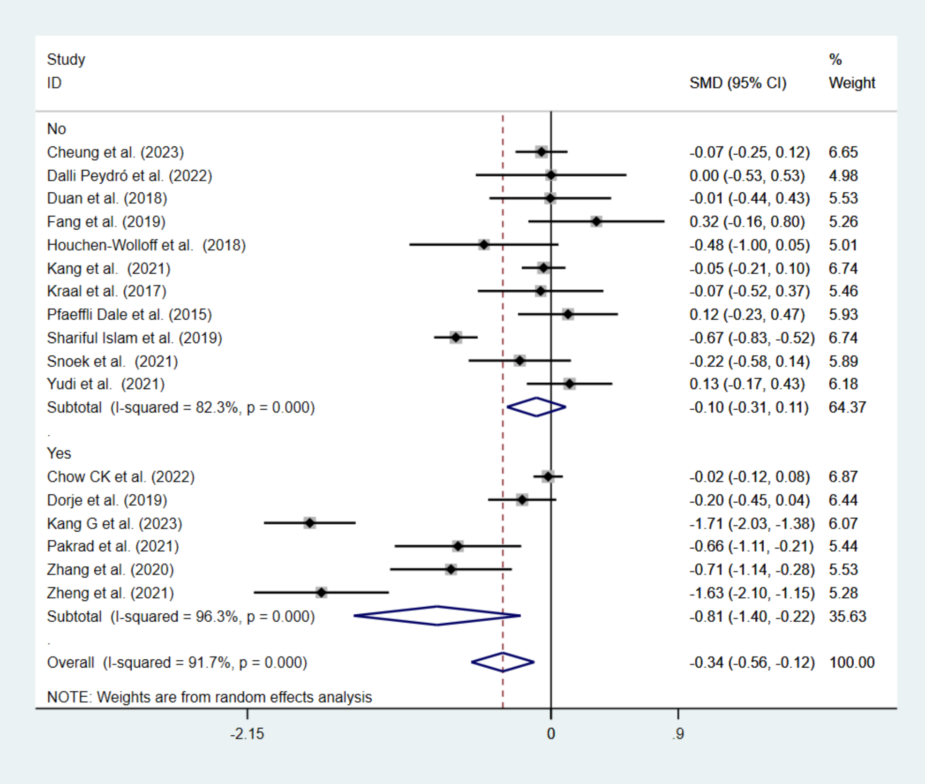

Subgroup analyses: Results stratified by different levels of adherence (higher a dherence rates-≥90% vs. lower adherence rates-<90%)

Figure S26. Mean effect sizes of quality of life

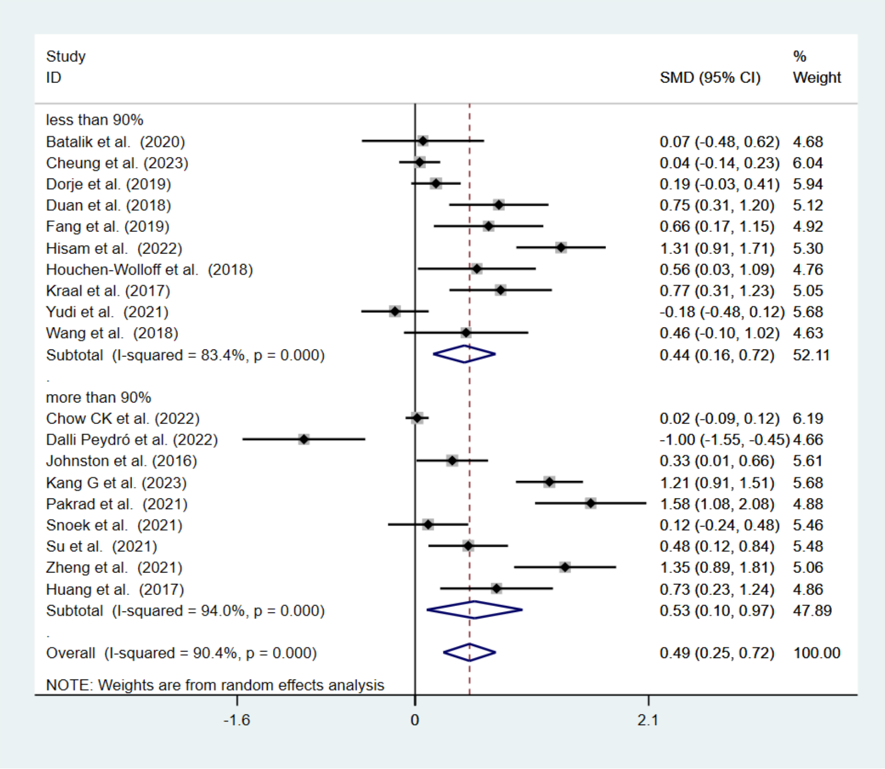

Figure S27. Mean effect sizes of anxiety

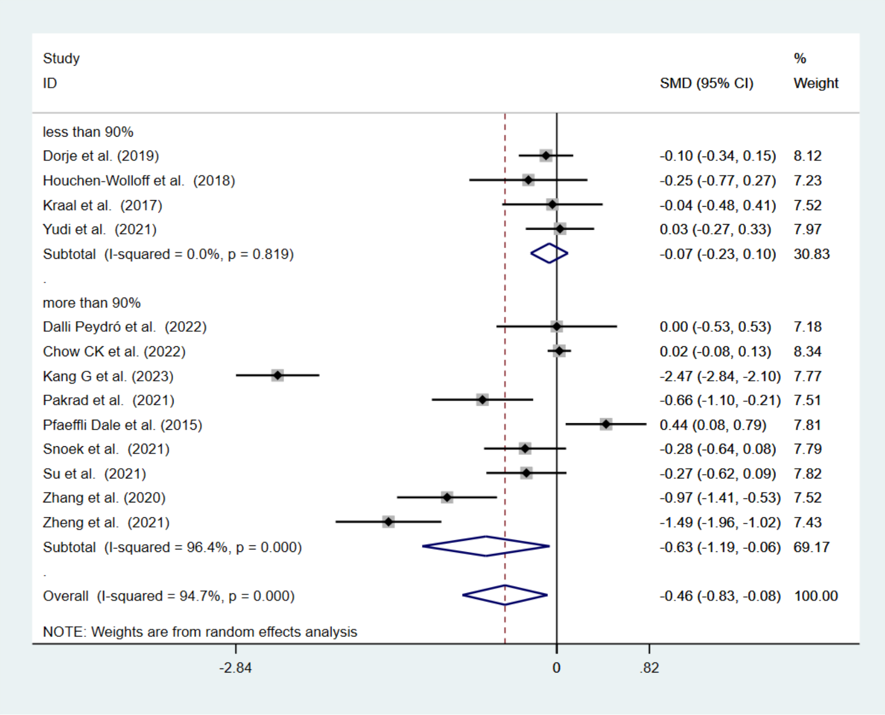

Figure S28. Mean effect sizes of depression

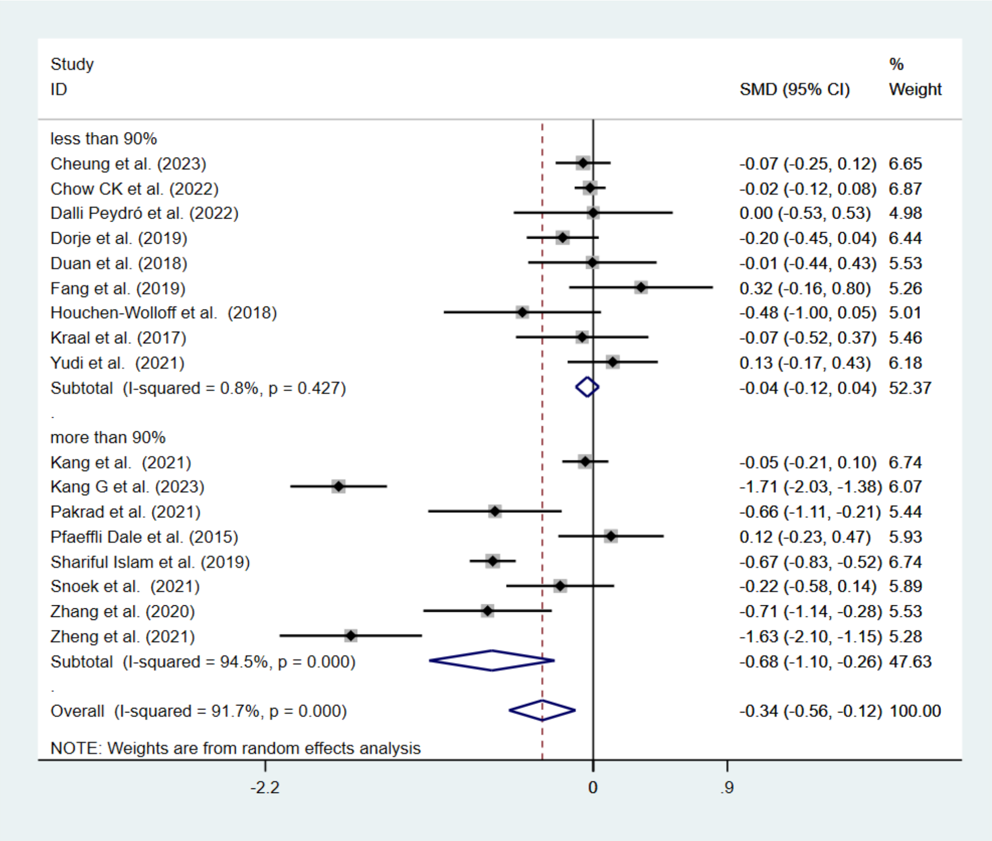

Searching strategies

Literature search in PubMed

No.	Search Details	Results
#1	((((("Coronary Disease"[Mesh] OR "Coronary Artery Disease"[Mesh]) OR "Myocardial Ischemia"[Mesh]) OR "Acute Coronary Syndrome"[Mesh]) OR "Coronary Artery Bypass"[Mesh]) OR "Myocardial Revascularization"[Mesh]) OR "Percutaneous Coronary Intervention"[Mesh]	507,428
#2	((((((((((((((((((((((((((((((((((Coronary Diseases[Title/Abstract]) OR (Disease*, Coronary[Title/Abstract])) OR (Coronary Heart Disease*[Title/Abstract])) OR (Disease*, Coronary Heart[Title/Abstract])) OR (Heart Disease*, Coronary[Title/Abstract])) OR (Ischemia*, Myocardial[Title/Abstract])) OR (Myocardial Ischemia*[Title/Abstract])) OR (Ischemic Heart Disease*[Title/Abstract])) OR (Heart Disease*, Ischemic[Title/Abstract])) OR (Disease*, Ischemic Heart[Title/Abstract])) OR (Artery Disease*, Coronary[Title/Abstract])) OR (Coronary Artery Disease*[Title/Abstract])) OR (Left Main Coronary Artery Disease[Title/Abstract])) OR (Left Main Disease*[Title/Abstract])) OR (Left Main Coronary Disease[Title/Abstract])) OR (Coronary Arterioscleros*[Title/Abstract])) OR (Coronary Atheroscleros*[Title/Abstract])) OR (Acute Coronary Syndrome*[Title/Abstract])) OR (Coronary Syndrome*, Acute[Title/Abstract])) OR (Syndrome*, Acute Coronary[Title/Abstract])) OR (Coronary Intervention*, Percutaneous[Title/Abstract])) OR (Intervention*, Percutaneous Coronary[Title/Abstract])) OR (Percutaneous Coronary Interventions[Title/Abstract])) OR (Percutaneous Coronary Revascularization*[Title/Abstract])) OR (Coronary Revascularization*, Percutaneous[Title/Abstract])) OR (Revascularization*, Percutaneous Coronary[Title/Abstract])) OR (Percutaneous Transluminal Coronary Angioplast*[Title/Abstract])) OR (Artery Bypass*, Coronary[Title/Abstract])) OR (Bypass*, Coronary Artery[Title/Abstract])) OR (Coronary Artery Bypasses[Title/Abstract])) OR (Coronary Artery Bypass Surgery[Title/Abstract])) OR (Coronary Artery Bypass Grafting[Title/Abstract])) OR (Aortocoronary Bypass*[Title/Abstract])) OR (Bypass*, Aortocoronary[Title/Abstract])) OR (Bypass Surgery, Coronary Artery[Title/Abstract])) OR (Myocardial Revascularizations[Title/Abstract])) OR (Revascularization*, Myocardial[Title/Abstract])) OR (Internal Mammary Artery Implantation[Title/Abstract])	278,233
#3	#1 OR #2	598,829
#4	((((((((("Telemedicine"[Mesh]) OR "Smartphone"[Mesh]) OR "Mobile Applications"[Mesh]) OR "Cell Phone"[Mesh]) OR "Electronic Mail"[Mesh]) OR "Internet-Based Intervention"[Mesh]) OR "Text Messaging"[Mesh]) OR "Distance Counseling"[Mesh]) OR "Reminder Systems"[Mesh]) OR "Telecommunications"[Mesh]) OR "Mobile Health Units"[Mesh]	136,282
#5	((Mobile Health[Title/Abstract]) OR (mHealth[Title/Abstract])) OR (Telehealth[Title/Abstract])) OR (eHealth[Title/Abstract])) OR (Smartphone*[Title/Abstract])) OR (Smart Phone*[Title/Abstract])) OR (Application*, Mobile[Title/Abstract])) OR (Mobile Application*[Title/Abstract])) OR (Mobile App[Title/Abstract])) OR (Mobile Apps[Title/Abstract])) OR (Portable Electronic App[Title/Abstract])) OR (Portable Electronic Apps[Title/Abstract])) OR (Electronic App, Portable[Title/Abstract])) OR (Portable Electronic Application*[Title/Abstract])) OR (Portable Software App[Title/Abstract])) OR (Portable Software	109,602

	Apps[Title/Abstract])) OR (Software App, Portable[Title/Abstract])) OR (Portable Software Application*[Title/Abstract])) OR (Software Application, Portable[Title/Abstract])) OR (Smartphone App[Title/Abstract])) OR (Smartphone Apps[Title/Abstract])) OR (Phone*, Cell[Title/Abstract])) OR (Cellular Phone*[Title/Abstract])) OR (Phone*, Cellular[Title/Abstract])) OR (Telephone*, Cellular[Title/Abstract])) OR (Cellular Telephone*[Title/Abstract])) OR (Cell Phone*[Title/Abstract])) OR (Portable Cellular Phone*[Title/Abstract])) OR (Cellular Phone*, Portable[Title/Abstract])) OR (Transportable Cellular Phone*[Title/Abstract])) OR (Cellular Phone*, Transportable[Title/Abstract])) OR (Mobile Phone*[Title/Abstract])) OR (Phone*, Mobile[Title/Abstract])) OR (Mobile Telephone*[Title/Abstract])) OR (Telephone*, Mobile[Title/Abstract])) OR (Car Phone*[Title/Abstract])) OR (Phone*, Car[Title/Abstract])) OR (Mail, Electronic[Title/Abstract])) OR (E-Mail*[Title/Abstract])) OR (E Mail[Title/Abstract])) OR (Email*[Title/Abstract])) OR (Internet Based Intervention[Title/Abstract])) OR (Internet-Based Intervention*[Title/Abstract])) OR (Intervention*, Internet-Based[Title/Abstract])) OR (Web-based Intervention*[Title/Abstract])) OR (Intervention*, Web-based[Title/Abstract])) OR (Web based Intervention[Title/Abstract])) OR (Online Intervention*[Title/Abstract])) OR (Intervention*, Online[Title/Abstract])) OR (Internet Intervention*[Title/Abstract])) OR (Intervention*, Internet[Title/Abstract])) OR (Messaging, Text[Title/Abstract])) OR (Texting*[Title/Abstract])) OR (Short Message Service[Title/Abstract])) OR (Text Message*[Title/Abstract])) OR (Message*, Text[Title/Abstract])) OR (Counseling, Distance[Title/Abstract])) OR (E-Therap*[Title/Abstract])) OR (E Therapy[Title/Abstract])) OR (E-Counseling[Title/Abstract])) OR (E Counseling[Title/Abstract])) OR (System*, Reminder[Title/Abstract])) OR (Telecommunication*[Title/Abstract])) OR (Telegraph*[Title/Abstract])) OR (Teleconference*[Title/Abstract])) OR (Health Unit*, Mobile[Title/Abstract])) OR (Mobile Health Unit*[Title/Abstract])) OR (Unit*, Mobile Health[Title/Abstract])) OR (Mobile Clinic*[Title/Abstract])) OR (Clinic*, Mobile[Title/Abstract])) OR (Mobile Health Van[Title/Abstract])) OR (Health Van, Mobile[Title/Abstract])) OR (Van, Mobile Health[Title/Abstract])) OR (Field Hospital*[Title/Abstract])) OR (Mobile Hospital*[Title/Abstract])) OR (Hospital*, Mobile[Title/Abstract])	
#6	#4 OR #5	203,630
#7	("Randomized Controlled Trial" [Publication Type]) OR ((randomized [Title/Abstract]) OR (placebo[Title/Abstract]))	1,007,642
#8	#3 AND #6 AND #7	448

Literature search in Embase

No.	Search Details	Results
#1	'coronary artery disease'/exp OR 'acute coronary syndrome'/exp OR 'coronary artery bypass graft'/exp OR 'percutaneous coronary intervention'/exp OR 'coronary diseases':ab,ti OR 'disease*, coronary':ab,ti OR 'coronary heart diseases':ab,ti OR 'disease*, coronary heart':ab,ti OR 'heart disease*, coronary':ab,ti OR 'ischemia*, myocardial':ab,ti OR 'artery disease*, coronary':ab,ti OR 'left main coronary artery disease':ab,ti OR 'left main disease*':ab,ti OR 'coronary arterioscleros*':ab,ti	529,378
#2	'telemedicine'/exp OR 'telemedicine' OR 'smartphone'/exp OR 'smartphone' OR 'mobile application'/exp OR 'mobile application' OR 'mobile phone'/exp OR 'mobile phone' OR 'web-based intervention'/exp OR	197,974

	'web-based intervention' OR 'text messaging'/exp OR 'text messaging' OR 'e-counseling'/exp OR 'e-counseling' OR 'telecommunication'/exp OR 'telecommunication'	
#3	'mobile health':ab,ti OR 'mhealth':ab,ti OR 'telehealth':ab,ti OR 'smartphone':ab,ti OR 'mobile application':ab,ti OR 'portable electronic app':ab,ti OR 'smartphone application':ab,ti OR 'mobile phone':ab,ti OR 'web-based intervention':ab,ti OR 'short message service':ab,ti OR 'text message':ab,ti OR 'telecommunication':ab,ti	66,271
#4	#2 OR #3	203,063
#5	'clinical trial'/de OR 'randomized controlled trial'/de OR 'randomization'/de OR 'single blind procedure'/de OR 'double blind procedure'/de OR 'crossover procedure'/de OR 'placebo'/de OR 'prospective study'/de OR 'randomi?ed controlled' NEXT/1 trial* OR rct OR 'randomly allocated' OR 'allocated randomly' OR 'random allocation' OR allocated NEAR/2 random OR single NEXT/1 blind* OR double NEXT/1 blind* OR (treble OR triple) NEAR/1 blind* OR placebo*	2,905,855
#6	#1 AND #4 AND #5	555

Literature search in Cochrane

No.	Search Details	Results
#1	Mesh descriptor: [Telemedicine] explode all trees	4,210
#2	(Mobile Health):ti,ab,kw OR (mHealth):ti,ab,kw OR (Telehealth):ti,ab,kw OR (eHealth):ti,ab,kw	18,703
#3	Mesh descriptor: [Smartphone] explode all trees	1,006
#4	Mesh descriptor: [Mobile Applications] explode all trees	1,538
#5	(Application*, Mobile):ti,ab,kw OR (Mobile Application*):ti,ab,kw OR (Mobile App*):ti,ab,kw OR (App*, Mobile):ti,ab,kw OR (Portable Electronic App*):ti,ab,kw	18,515
#6	Mesh descriptor: [Cell Phone] explode all trees	3,099
#7	(Phone*, Cell): ti,ab,kw OR (Cellular Phone*):ti,ab,kw OR (Phone*, Cellular):ti,ab,kw OR (Telephone*, Cellular):ti,ab,kw OR (Cellular Telephone*):ti,ab,kw	313
#8	Mesh descriptor: [Internet-Based Intervention] explode all trees	546
#9	(Internet Based Intervention):ti,ab,kw OR (Internet-Based Intervention*):ti,ab,kw OR (Intervention*, Internet-Based):ti,ab,kw OR (Web-based Intervention*):ti,ab,kw OR (Intervention*, Web-based):ti,ab,kw	11,860
#10	Mesh descriptor: [Text Messaging] explode all trees	1,483
#11	(Messaging, Text):ti,ab,kw OR (Texting*):ti,ab,kw OR (Short Message Service):ti,ab,kw OR (Text Message*):ti,ab,kw OR (Message*, Text):ti,ab,kw	6,714
#12	Mesh descriptor: [Telecommunications] explode all trees	10,731
#13	#1 or #2 or #3 or #4 or #5 or #6 or #7 or #8 or #9 or #10 or #11 or #12	48,800
#14	Mesh descriptor: [Coronary Disease] explode all trees	18,357
#15	(Coronary Diseases):ti,ab,kw OR (Disease*, Coronary):ti,ab,kw OR (Coronary Heart Disease*):ti,ab,kw OR (Disease*, Coronary Heart):ti,ab,kw OR (Heart Disease*, Coronary):ti,ab,kw	41,200
#16	Mesh descriptor: [Myocardial Ischemia] explode all trees	36,843
#17	(Ischemia*, Myocardial):ti,ab,kw OR (Ischemic Heart Disease*):ti,ab,kw OR (Heart Disease*, Ischemic):ti,ab,kw OR (Disease*, Ischemic Heart):ti,ab,kw	11,650

#18	Mesh descriptor: [coronary artery disease] explode all trees	9,369
#19	Mesh descriptor: [Acute Coronary Syndrome] explode all trees	3,273
#20	Mesh descriptor: [Percutaneous Coronary Intervention] explode all trees	8,381
#21	(Coronary Intervention*, Percutaneous):ti,ab,kw OR (Intervention*, Percutaneous Coronary):ti,ab,kw OR (Percutaneous Coronary Revascularization*):ti,ab,kw OR (Coronary Revascularization*, Percutaneous):ti,ab,kw OR (Revascularization*, Percutaneous Coronary):ti,ab,kw OR (Percutaneous Transluminal Coronary Angioplast):ti,ab,kw	4,560
#22	Mesh descriptor: [Coronary Artery Bypass] explode all trees	6,229
#23	(Artery Bypass*, Coronary):ti,ab,kw OR (Coronary Artery Bypass Surgery):ti,ab,kw OR (Coronary Artery Bypass Grafting):ti,ab,kw OR (Bypass*, Aortocoronary):ti,ab,kw OR (Bypass Surgery, Coronary Artery):ti,ab,kw	13,365
#24	Mesh descriptor: [Myocardial Revascularization] explode all trees	10,681
#25	#14 OR #15 OR #16 OR #17 OR #18 OR #19 OR #20 OR #21 OR #22 OR #23 OR #24	68,913
#26	(Randomized controlled trial) OR ("randomized-controlled trials") OR (Random allocation) OR (Double blind method) OR (Single blind method)	1,117,207
#27	#13 AND #25 AND #26	1,110

Literature search in Web of Science

No.	Search Details	Results
#1	TS=(telemedicine OR smartphone* OR Mobile Application* OR cellphone OR Electronic Mail OR Internet-Based Intervention* OR Text Messaging OR Distance Counseling OR Reminder Systems OR Telecommunication* OR mobile health units OR mobile health OR mhealth OR Telehealth OR eHealth OR mobile app* OR Portable Electronic App* OR Portable Electronic Application* OR Portable Software App* OR Portable Software Application* OR Smartphone App* OR Cellular Phone* OR Cellular Telephone* OR Cell Phone* OR Portable Cellular Phone* OR Transportable Cellular Phone* OR Mobile Phone* OR Mobile Telephone* OR Car Phone* OR E-Mail* OR Web-based Intervention* OR Online Intervention* OR Internet Intervention* OR Texting* OR Short Message Service OR Text Message*)	2,412,389
#2	TS=(Coronary Disease* OR myocardial ischemia OR Coronary Artery Disease OR Acute Coronary Syndrome OR Percutaneous Coronary Revascularization OR percutaneous coronary intervention OR coronary artery bypass OR Myocardial Revascularization OR Disease*, Coronary OR Coronary Heart Disease* OR Ischemia*, Myocardial OR Ischemic Heart Disease* OR Coronary Artery Disease* OR Left Main Coronary Artery Disease OR Left Main Disease* OR Left Main Coronary Disease OR Coronary Arterioscleros* OR Coronary Atheroscleros* OR Coronary Syndrome*, Acute OR Coronary Intervention*, Percutaneous OR Percutaneous Coronary Revascularization* OR Percutaneous Transluminal Coronary Angioplast* OR Artery Bypass*, Coronary OR Coronary Artery Bypass Surgery OR Coronary Artery Bypass Grafting OR Aortocoronary Bypass* OR Bypass Surgery, Coronary Artery OR Internal Mammary Artery Implantation OR Revascularization*, Myocardial)	1,020,717
#3	TS= (Randomized Controlled Trial OR randomized OR placebo OR clinical trial* OR controlled trial* OR single blind* OR double blind*)	2,564,953
#4	#1 AND #2 AND #3	1,484

Literature search in CINAHL

No.	Search Details	Results
#1	(telemedicine OR smartphone* OR Mobile Application* OR cellphone OR Electronic Mail OR Internet-Based Intervention* OR Text Messaging OR Distance Counseling OR Reminder Systems OR Telecommunication* OR mobile health units OR mobile health OR mhealth OR Telehealth OR eHealth OR mobile app* OR Portable Electronic App* OR Portable Electronic Application* OR Portable Software App* OR Portable Software Application* OR Smartphone App* OR Cellular Phone* OR Cellular Telephone* OR Cell Phone* OR Portable Cellular Phone* OR Transportable Cellular Phone* OR Mobile Phone* OR Mobile Telephone* OR Car Phone* OR E-Mail* OR Web-based Intervention* OR Online Intervention* OR Internet Intervention* OR Texting* OR Short Message Service OR Text Message*)	430,663
#2	(Coronary Disease* OR myocardial ischemia OR Coronary Artery Disease OR Acute Coronary Syndrome OR Percutaneous Coronary Revascularization OR percutaneous coronary intervention OR coronary artery bypass OR Myocardial Revascularization OR Disease*, Coronary OR Coronary Heart Disease* OR Ischemia*, Myocardial OR Ischemic Heart Disease* OR Coronary Artery Disease* OR Left Main Coronary Artery Disease OR Left Main Disease* OR Left Main Coronary Disease OR Coronary Arterioscleros* OR Coronary Atheroscleros* OR Coronary Syndrome*, Acute OR Coronary Intervention*, Percutaneous OR Percutaneous Coronary Revascularization* OR Percutaneous Transluminal Coronary Angioplast* OR Artery Bypass*, Coronary OR Coronary Artery Bypass Surgery OR Coronary Artery Bypass Grafting OR Aortocoronary Bypass* OR Bypass Surgery, Coronary Artery OR Internal Mammary Artery Implantation OR Revascularization*, Myocardial)	938,583
#3	(Randomized Controlled Trial OR randomized OR placebo OR clinical trial* OR controlled trial* OR single blind* OR double blind*)	2,136,015
#4	#1 AND #2 AND #3	1,608
